# Recent Advances in Smart Stimulus-Responsive Hydrogels for Precision Drug Delivery in Tumours

**DOI:** 10.3390/gels12020098

**Published:** 2026-01-23

**Authors:** Huiling Zuo, Yuhang Jiao, Jiaxin Chen, Sen Tong, Yan Li, Wei Zhao

**Affiliations:** 1 Yunnan Key Laboratory of Integrated Traditional Chinese and Western Medicine for Chronic Disease in Prevention and Treatment, Yunnan University of Chinese Medicine, Kunming 650500, China; zuohuiling@ynucm.edu.cn (H.Z.); jiaoyuhang@ynucm.edu.cn (Y.J.); chenjiaxin@ynucm.edu.cn (J.C.); tongsen@ynucm.edu.cn (S.T.); 2 Faculty of Life Science and Technology, Kunming University of Science and Technology, Kunming 650500, China; liyanken@126.com

**Keywords:** hydrogel, stimulus-responsive type, drug delivery, tumour

## Abstract

Cancer remains one of the most prominent global health concerns, posing a substantial threat to public health. Millions of people die from cancer each year, and many cancer types remain incurable at present. Conventional cancer treatments, including surgery, chemotherapy, radiotherapy, and immunotherapy, often fail to achieve optimal clinical outcomes and are frequently associated with severe trauma and adverse effects. Consequently, there is an urgent need to develop novel therapeutic strategies to address these limitations. Hydrogels have been widely utilised as platforms for loading drugs, proteins, DNA, and stem cells in biomedical tissue repair and cancer therapy. Through modification of their physicochemical properties and functions, hydrogels can be endowed with responsiveness to multiple stimuli. In recent years, stimuli-responsive hydrogels (also known as smart-responsive hydrogels), as novel drug delivery systems, have demonstrated remarkable efficacy in cancer treatment. Stimuli-responsive hydrogels are capable of altering their mechanical properties, swelling behaviour, hydrophilicity, bioactivity, and molecular permeability in response to endogenous stimuli (including pH, ROS, and temperature) and exogenous stimuli (including light, ultrasound, and magnetic fields). This review highlights recent advances and applications of responsive hydrogels triggered by endogenous stimuli (including pH, ROS, and temperature) and exogenous stimuli (including light, ultrasound, and magnetic force) in cancer drug delivery and treatment. Finally, the current application limitations and future prospects of smart-responsive hydrogels are summarised.

## 1. Introduction

Currently, cancer represents one of the most significant global health challenges and poses a major threat to public health. Millions of people die from cancer each year, and many cancer types remain incurable at present [1]. Conventional cancer treatments primarily include surgery, chemotherapy, and radiotherapy [2]. Although these approaches can, to some extent, control tumour progression, each has its own inherent limitations. Surgery is often effective for early-stage cancers; however, it cannot completely eradicate tumour dissemination and metastasis in mid- to late-stage disease and may increase the risk of recurrence [3]. Chemotherapy disrupts cell division and induces apoptosis through cytotoxic drugs, but its lack of specificity frequently results in damage to normal proliferating cells [4]. Moreover, long-term chemotherapy may lead to the development of drug resistance in tumour cells, thereby reducing therapeutic efficacy [4]. Radiotherapy eradicates cancer cells through ionising radiation but inevitably causes collateral damage to the surrounding healthy tissues [5]. Consequently, there is an urgent need to develop novel therapeutic strategies to overcome these challenges. The tumour microenvironment (TME) refers to the non-cancerous cells and components within a tumour, including the molecules they produce and secrete [6]. Continuous interactions between tumour cells and the TME play a decisive role in tumourigenesis, progression, metastasis, and therapeutic response [7]. Compared with normal tissues, tumour tissues exhibit a range of distinctive characteristics, including lower pH values, local hypoxia, upregulated reduced-glutathione (GSH) expression, higher hydrogen peroxide (H_2_O_2_) concentrations, and elevated ATP levels [8]. On the basis of these features, smart responsive nanomedicine delivery systems can be designed to enhance the efficiency of targeted tumour therapy.

Hydrogels are three-dimensional networks of hydrophilic polymers with a wide range of biomedical applications [9]. In biomedicine, hydrogels are extensively used in tissue regeneration, bioimaging, biosensors, investigations of physiological and pathological mechanisms, and local drug delivery systems (DDS) [10]. Modern hydrogels are characterised by high water content, excellent biocompatibility, tunable mechanical properties, and dynamic responsiveness to environmental stimuli (such as pH, temperature, light, and enzymes) (Table 1) [11]. Drug loading capacity and encapsulation efficiency are highly dependent on the material structure and chemical composition of hydrogels [12,13,14]. More importantly, their sensitivity to the TME directly determines drug release kinetics [15]. Based on these properties, responsive hydrogels have been developed to deliver active pharmaceutical ingredients efficiently to target sites, reduce drug-induced side effects, and maximise therapeutic efficacy [16]. Smart-responsive hydrogels not only release drugs in response to TME-related stimuli but can also undergo degradation and clearance triggered by microenvironmental conditions [17]. This behaviour enhances the biocompatibility of nanomaterials and reduces the risk of long-term retention. In biomedicine, hydrogels are generally classified into two main categories: natural hydrogels [e.g., nucleic acids, chitosan (CS), guar gum, cellulose, hyaluronic acid (HA), and alginate] and synthetic hydrogels [e.g., poly(acrylic acid) (PAA), poly(acrylamide) (PAM), poly(vinyl alcohol) (PVA), polyethylene glycol (PEG), poly(vinyl pyrrolidone) (PVP), and poly(lactic acid) (PLA)] [18]. Natural hydrogels are typically non-toxic, low-cost, abundant, and highly biocompatible and biodegradable [19]. These materials can effectively mimic the extracellular matrix microenvironment, exhibit good affinity with biological tissues, and generate degradation products that are usually natural metabolites, thereby eliciting minimal immune rejection. The abundance of hydroxyl, carboxyl, and amino groups in composite hydrogels also facilitates the loading of various therapeutic agents [20]. However, their biocompatibility and immunogenicity issues cannot be overlooked. By contrast, composite hydrogels based on synthetic polymers, such as PEG and PVA, allow for better control over mechanical and physicochemical properties. Nevertheless, residual catalysts or chemical reagents from the synthesis process may persist within the polymers, and the accumulated degradation products may induce local inflammatory responses [21]. Surface modification strategies can be employed to improve biocompatibility and reduce immunogenicity [22]. Furthermore, the integration of natural and synthetic polymers into composite hydrogels enables a balance between biocompatibility and mechanical performance. Notably, the performance and functionality of smart hydrogels fundamentally depend on their chemical composition and synthesis methods [23].

During cancer treatment, therapeutic modalities, such as surgery, radiotherapy, chemotherapy, and immunotherapy, frequently face challenges, including limited efficacy, pronounced side effects, and the emergence of drug resistance in tumour cells [24]. Smart-responsive hydrogels can serve as key delivery vehicles to address these limitations. By improving drug utilisation, they reduce ineffective consumption resulting from off-target effects and minimise damage to healthy tissues caused by chemotherapeutic agents, thereby significantly enhancing therapeutic outcomes [25]. Although many reviews have focused on the synthesis, characterisation, and physicochemical properties of hydrogels, as well as their development as DDS, this review places particular emphasis on their responsiveness—specifically, their capacity to respond to the TME and external stimuli. Through rapid sol–gel phase transitions or in situ chemical polymerisation, these responsive hydrogels enable targeted drug delivery and controlled release, ultimately contributing to improved tumour treatment outcomes.

**Table 1:** Single-stimulus-responsive hydrogels.

NO.	Hydrogel	Response Type (Single-Stimulus)	Mechanism	Tumour Type	Ref.
1	aP/IR@FMKB	Enzyme	The hydrogel responds to MMP-2 (matrix metalloproteinase-2) enzymes in the TME and dissociates, releasing the encapsulated drug to exert antitumour and anti-immunosuppressive effects. This system exhibits outstanding photothermal properties, enabling prolonged retention at the tumour site and significantly inhibiting the growth of primary, distant, and recurrent tumours.	4T1 murine breast tumour cells	[26]
2	UCNJ	ATP	UCNJs can recognise ATP in tumour cells, leading to hydrogel degradation and DOX release.	HeLa cells	[27]
3	NHS–SS–NHS crosslinked chitosan nano-hydrogels	GSH	The study successfully synthesised GSH-responsive chitosan nano-hydrogels using the active ester method. This carrier efficiently released DOX (>80%) under 10 mM GSH conditions and demonstrated superior antitumor activity against A549 cells, particularly in high GSH environments.	A549 cells	[28]
4	DH-G3-CPT	PH	The DH-G3-CPT drug delivery system possesses both injectability and sustained drug release capabilities. Its hydrogel drug delivery system, loaded with camptothecin (CPT), releases the drug through ester hydrolysis and prolongs the release duration. Additionally, the self-cleavage release kinetics of camptothecin are influenced by pH levels.	Head and neck cancer model of mouse	[29]
5	AHB Gel	ATP	AHB Gel is a novel TME-targeted DNA hybrid hydrogel for ATP-based fluorescence imaging. Upon ATP exposure, AHB Gel rapidly emits fluorescence within 3 min, with the signal appearing exclusively at sites exposed to high ATP concentrations. This results in a sharp boundary between ATP-rich and ATP-poor regions.	Tumour	[30]
6	PN-DATKC2/PGNR-C3 hydrogel	ROS	The gel state is achieved through a phase transition at 37 °C body temperature. The ROS-responsive nanocomposite hydrogel serves both as an accurate drug delivery platform and as a combined cancer treatment system for localised PDT and PTT.	HeLa cells	[31]
7	Au NBPs & Pt NCs @ DOX gel	Light	The hydrogel exhibited rapid gelation and excellent injectability. Attributed to the high absorbance of Au NBPs, the nanocomposite hydrogels revealed superior photothermal effect under NIR irradiation, and the DOX release was also regulated by NIR laser. In addition, the catalase- and peroxidase-like activities of Pt NCs were validated to convert endogenous H_2_O_2_ into ROS and O_2_ in situ to achieve chemodynamic therapy (CDT) and alleviate the hypoxic microenvironment. Simultaneously, catalytic therapy combined with NIR irradiation exhibited the strongest inhibition to the growth of 4T1 tumour in vitro and in vivo.	4T1 tumour	[32]
8	TNP/DOX/ZnPC	Thermal	The thermal-responsive nanoparticles (TNPs) were prepared by the nanoprecipitation technology. Cell inhibition showed that the best cell inhibition was found, with cell viability of 18.5%, when the weight ratio of DOX and ZnPC encapsulated in the TNP reached about 1:5.	5637 cells; nude mice bearing 5637 cells	[33]
9	HiROSponse DOX/PTX	ROS	HiROSponse is loaded with the two cytostatic drugs (hiROSponse DOX/PTX): doxorubicin (DOX) and paclitaxel (PTX). DOX release is mainly controlled by Fickian diffusion. In a syngeneic malignant melanoma-bearing mouse model, hiROSponse DOX/PTX slows tumour growth without causing adverse side effects and doubles the relative survival probability.	Melanoma-bearing mouse model	[34]

## 2. Drug Release Mechanism of Hydrogels

Hydrogel DDS exhibit excellent biocompatibility, biodegradability, and the capacity for controlled drug release [35]. The mechanisms governing drug release from hydrogels can be broadly classified into two categories: Fickian diffusion [36] and chemically controlled release. Crosslinking within hydrogels may be either physical or chemical. Physical crosslinking is formed through secondary interactions, such as hydrogen bonding between polar groups on polymer chains, whereas chemical crosslinking is achieved via covalent bonds between different functional groups on polymer chains using specific crosslinking agents [18].

### 2.1. Fickian Diffusion

When interactions between the drug and the hydrogel matrix are weak, drug release predominantly follows Fick’s law of diffusion [37]. Drug molecules spontaneously diffuse from regions of higher concentration within the gel to the surrounding environment of lower concentration [38]. In this case, the release rate is determined entirely by the diffusion rate of the drug within the hydrogel, while the gel structure itself remains essentially unchanged during the release process. A higher swelling degree leads to a looser network structure and more accessible diffusion pathways. Consequently, this diffusion mechanism is typically characterised by a rapid initial release followed by a gradual decrease in the release rate. The majority of pH-responsive hydrogels exhibit drug release behaviour governed by Fickian diffusion. For example, Meena et al. developed a pH-responsive hydrogel for sodium diclofenac delivery, in which the gels containing sodium diclofenac exhibited prolonged drug release at pH 7.4, with release kinetics following Fickian diffusion [39]. Similarly, Siddiqua prepared a pH-sensitive pectin/acrylamide hydrogel for targeted colon drug delivery, and the drug release mechanism also followed Fickian diffusion [40]. In addition, it has been observed that the swelling capacity of hydrogels influences their drug release behaviour. Zhao et al. reported that composite hydrogels composed of sodium alginate and cellulose nanocrystals with high swelling capacity did not follow Fickian diffusion with respect to drug release kinetics [41].

### 2.2. Chemically Controlled Release

In contrast to Fickian diffusion, smart-responsive hydrogels primarily achieve drug loading and release through the formation and cleavage of reversible or irreversible chemical bonds [42]. In this release mechanism, drugs are covalently linked to polymer chains via cleavable bonds, such as hydrogen bonds, ester bonds, amide bonds, or hydrazone bonds [43]. Drug release in its active form occurs only when specific environmental stimuli (such as particular pH conditions, enzymes, or reductants) trigger the cleavage of these chemical bonds. This strategy enables “zero-order release” (i.e., constant rate release), thereby effectively avoiding burst release and prolonging the duration of drug action [44,45,46]. Drugs are often encapsulated within biodegradable polymers, such as PLGA, CS, HA, and others [47]. The degradation rate of the hydrogel network directly influences the drug release rate. Hydrogel degradation may proceed via surface erosion (layer-by-layer degradation from the exterior inward) or bulk erosion (simultaneous degradation throughout the entire network) [48]. A representative example is enzyme-responsive hydrogels (ERHs), which degrade selectively in the presence of specific enzymes that are pathologically overexpressed at tumour or inflammatory sites, thereby enabling targeted drug release [49]. Photoresponsive hydrogels facilitate hydrogel degradation and drug release through photoisomerisation, photodegradation, or photothermal effect-induced structural changes or network disruption [50]. Collectively, these stimulus-induced physical and chemical changes provide the molecular basis for on-demand and precise drug release from nanocomposite hydrogels.

## 3. Endogenous Stimulants

### 3.1. pH-Responsive Type

Within the TME, cancer cells primarily rely on a combination of glycolysis, oxidative phosphorylation, and other metabolic pathways to generate energy, producing acidic metabolic by-products in the form of CO_2_ and H^+^ [51]. Compared with normal tissues, the pH of solid tumours is therefore relatively low. The swelling behaviour of hydrogels is highly sensitive to physicochemical factors such as pH [52]. By exploiting this property, pH-sensitive hydrogels can respond to the acidic microenvironment of solid tumours, thereby triggering targeted drug release [53]. Andrade et al. developed a novel pH-responsive hydrogel by adjusting the ratio of CS and alginate polymers to polyvinyl alcohol. This hydrogel exhibited high swelling capacity under acidic pH conditions, enabling relatively rapid drug release [54].

**Figure 1:**
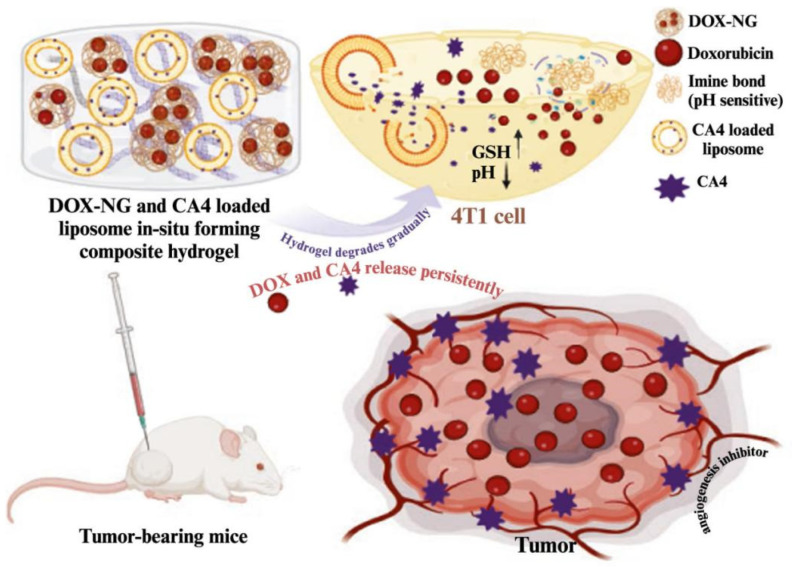
Describes the synthesis and working mechanism of DOX loaded pH-sensitive nanogels and CA4 loaded liposomes. GSH ↑, high level GSH; pH ↓, Low pH value [58].

A wide range of nanoscale DDS has been investigated for pH responsiveness, including liposomes, micelles, hydrogels, dendrimers, organic–inorganic hybrid nanoparticles, and microspheres at the micrometre scale [55]. Prodrugs delivered via pH-sensitive nanoscale DDS have been applied in cancer therapy and diagnostics, as well as in the treatment of inflammation, antimicrobial infections, and neurological disorders [56]. pH-sensitive natural polymers include CS, alginates, heparin, HA, and cellulose derivatives [57]. Owing to their favourable biocompatibility, availability, and tunability, these polymers are widely used in hydrogel fabrication. Saba et al. developed a composite hydrogel system integrating doxorubicin (DOX) loaded pH-sensitive nanogels and combretastatin-A4 (CA4) loaded liposomes to achieve antitumour efficacy and prevent metastasis. The encapsulation efficiencies of DOX and CA4 reached 98 ± 1.15% and 87 ± 1.2%, respectively [58]. Experimental results demonstrated that CA4 loaded liposomes and DOX loaded nanogels effectively inhibited the proliferation of the 4T1 cell line and exhibited a synergistic effect when used in combination. In vivo studies further confirmed that, compared with other treatment groups in a 4T1 breast tumour mouse model, the composite hydrogel significantly suppressed tumour growth and prolonged survival time (Figure 1) [58].

**Figure 2:**
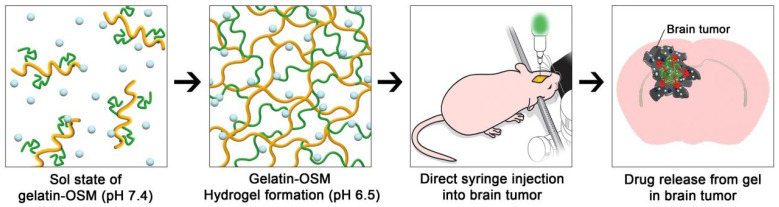
Solution–gel transformation of gelatin-OSM hydrogels under pH-stimulated sources and the continuous release of the drug [62].

Self-assembled peptide hydrogels have been shown to address the limitations of conventional chemotherapeutic agents, such as poor solubility, low selectivity, and severe adverse effects, and are therefore widely employed as drug delivery carriers [59]. Mechanistically, these peptide hydrogels improve selectivity through pH-responsive behaviour: they form stable gel structures under physiological conditions (pH 7.4) but undergo disassembly in the acidic TME (pH 5.8–6.5), accompanied by morphological transitions from nanofibres to nanospheres that enhance cellular drug uptake. In addition, protonation of basic amino acid residues under acidic conditions generates positive surface charges on peptide assemblies, promoting electrostatic interactions with negatively charged tumour cell membranes and thereby enhancing tumour-targeting selectivity [59]. Furthermore, injectable peptide hydrogels mitigate adverse reactions by forming localised “drug reservoirs” at peritumoural sites, enabling sustained drug release that reduces systemic exposure and minimises off-target toxicity to healthy tissues [59]. Yang et al. successfully developed a pH-triggered dynamic organic nanocomposite hydrogel in which a lipophilic mixture enhanced drug escape from the hydrogel, tumour penetration, and pH-responsive prolonged and sustained drug release [60]. These favourable physicochemical properties significantly inhibited tumour growth while reducing adverse effects on normal tissues, highlighting the considerable potential of pH-responsive dynamic organic nanocomposite hydrogels for clinical local tumour therapy [60]. For sustained-release formulations, excessively rapid drug release may lead to high local or systemic drug accumulation and associated side effects, whereas insufficient drug concentrations may be rapidly cleared by the bloodstream, thereby compromising antitumour efficacy [61]. Kang et al. reported that gelatin-OSM hydrogels exhibit pH-specific sol–gel transitions under in vivo conditions. Oligosulfamethazine (OSM) is a pH-responsive oligomer that is soluble at high pH (>7.4) but insoluble within the TME (pH 5.6–6.8) (Figure 2). This property enables gelatin–OSM hydrogels to maintain a gel state for a defined period, thereby allowing for sustained drug release [62]. Zhou et al. further demonstrated that a nanocomposite hydrogel (Col-APG-Cys@HHD) disintegrates under acidic conditions and releases drugs in response to a pH gradient (6.5–5.5). In vivo experiments showed that the Col-APG-Cys@HHD hydrogel effectively prevented peritoneal adhesion, inhibited tumour growth, and induced minimal side effects [63].

### 3.2. Temperature-Sensitive Hydrogels

Temperature-sensitive hydrogels are a class of smart biomaterials that exploit the synergistic interaction between the phase-transition behaviour of polymer chains and external temperature stimuli [64]. Through their crosslinked polymer networks, these hydrogels can swell in aqueous environments and retain substantial amounts of water. When exposed to changes in ambient temperature, they undergo volumetric alterations or phase transitions [65]. According to their phase-transition mechanisms, temperature-sensitive hydrogels are generally classified into two categories: those that exhibit gel-to-liquid transitions when the temperature exceeds a critical threshold, and those that undergo liquid-to-gel transitions when the temperature falls below a critical threshold [66]. The reversible volumetric changes or sol–gel transitions induced by temperature variations confer temperature-sensitive hydrogels with broad and potentially multifunctional applications [67].

Temperature-sensitive hydrogels commonly undergo sol–gel transitions at temperatures close to physiological body temperature (37 °C) [68]. Under thermal regulation, such hydrogels can function as “drug reservoirs”, enabling sustained drug release. A thermosensitive nanocarbon hydrogel (CS/GP@CN) developed by Tan et al. remains in a liquid state at room temperature and undergoes gelation at 37 °C, making it an effective platform for localised drug delivery [69]. Huo’s research group developed a thermosensitive phase-change hydrogel, termed Topo-Gel, which is injected into tumour tissue and subsequently transforms from a liquid into a gel at near-body temperature. This hydrogel enables sustained release of anticancer agents within tumour tissue, thereby maintaining prolonged antitumour activity [70]. In addition, Meng’s research team designed a temperature-sensitive tamoxifen phase-change hydrogel (Tam-Gel) for sustained drug delivery in breast cancer therapy. In a subcutaneous xenograft model established using MCF-7 cells in nude mice, Tam-Gel was directly injected into tumour sites. The results demonstrated sustained tamoxifen release within breast cancer tissue, leading to prolonged antitumour effects [71]. Li et al. further developed a temperature-sensitive nanocomposite supramolecular hydrogel, from which encapsulated DOX was released upon temperature elevation to 42 °C [72]. Collectively, the use of temperature-sensitive hydrogels to achieve in situ gelation and sustained intratumoural drug release represents an effective strategy to increase local drug concentration, prolong therapeutic duration, and enhance antitumour efficacy.

### 3.3. ATP-Responsive Hydrogels

**Figure 3:**
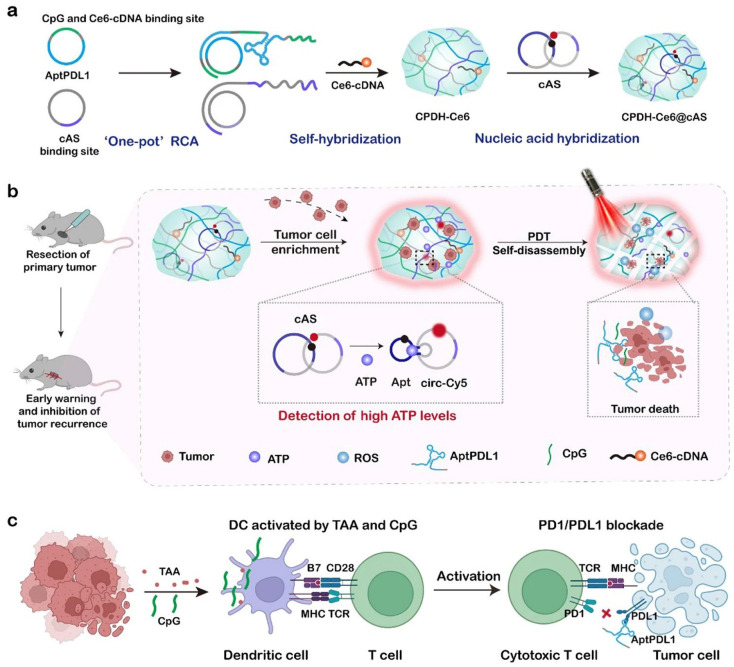
Synthesis and mechanism of action of ATP-responsive DNA hydrogels. (**a**) To construct a postoperative embedded hydrogel with integrated diagnostic and immunotherapy properties, we designed two types of DNA templates. One contains complementary sequences of PDL1 aptamer and CpG, and the other contains complementary sequences of tumor recurrence sensor binding sites. These two types of circular DNA templates and the photosensitizer Ce6-cDNA are used to synthesize light-responsive immunomodulatory hydrogels (CPDH-Ce6) by one-pot rolling circle amplification (RCA) reaction. The circular ATP sensor (cAS) was then loaded onto CPDH-Ce6 by sequence hybridization, resulting in CPDH-Ce6@cAS. (**b**) After primary tumor resection, CPDH-Ce6@cAS was locally embedded. Fluorescence recovery of cAS indicated tumor recurrence. Under external laser irradiation, CPDH-Ce6@cAS exerted photodynamic therapy and generated ROS to induce the hydrogel to release PDL1 aptamer and CpG. (**c**) The released CpG and TAA jointly promote DC maturation and T cell activation, and AptPDL1 blocks the binding of PD1 and PDL1 proteins to inhibit immune checkpoints and promote immune pathways to kill tumor cells [75].

In solid tumours, pro-inflammatory signals such as cell death, metabolic stress, and/or hypoxia within the tumour core stimulate active ATP export. This process results in the massive release of ATP into the extracellular space surrounding cancer cells, leading to significantly higher extracellular ATP levels in tumours compared with healthy tissues [73]. Accordingly, increasing attention has been directed towards the development and application of ATP-responsive nanohydrogels. Numerous studies have demonstrated that ATP-responsive nanohydrogels are widely employed in tumour cell diagnosis, imaging, and drug delivery [74]. Gao et al. designed a DNA nanohydrogel (DNH) to enable ATP-responsive near-infrared II (NIR-II) tumour imaging [1]. Moreover, ATP-responsive DNA nanohydrogels have achieved fluorescence imaging and selective cancer cell killing by exploiting differences in ATP levels between normal and tumour cell lines [74]. DNA hydrogels can be used not only for fluorescent tumour imaging but also as carriers for anticancer drugs such as DOX. In the presence of ATP, DNA hydrogels undergo cleavage, resulting in the release of anticancer agents and subsequent inhibition of tumour cell growth. However, postoperative tumour recurrence remains a major challenge in anticancer therapy. To address this issue, Wang et al. developed an ATP-responsive DNA hydrogel that responds to ATP levels within the TME, enabling timely detection of postoperative tumour recurrence and suppression of tumour cell growth [75]. Specifically, the PD-L1 aptamer incorporated into the DNA hydrogel captures and enriches in situ recurrent tumour cells, thereby increasing local ATP concentrations and rapidly generating warning signals. Upon detection of positive signals, localised laser irradiation can be applied to induce photodynamic therapy (PDT), leading to the eradication of captured tumour cells and the release of tumour-associated antigens, ultimately inhibiting tumour growth (Figure 3) [75].

Beyond their application in early tumour diagnosis, ATP-responsive hydrogels are predominantly utilised for anticancer drug delivery. High levels of ATP in tumour cells can induce changes in the chemical bonds/structures of hydrogels, thereby promoting drug release [27]. Chen and Liao’s research team developed DOX-loaded metal–organic framework nanoparticles (NMOFs) coated with stimuli-responsive nucleic acid polyacrylamide hydrogels. In cancer cells with high levels of ATP, the hydrogel coating degrades through the formation of ATP–aptamer complexes, resulting in DOX release and selective, efficient tumour cell killing. Importantly, compared with nucleic acid-gated NMOFs, hydrogel-encapsulated NMOFs exhibit a substantially higher drug loading capacity and effectively overcome the non-specific drug leakage observed in nucleic acid-protected NMOFs [76]. In addition, Sun’s group developed an ATP-responsive hydrogel capable of releasing immunoadjuvants in response to repeated chemotherapy or radiotherapy. Following intratumoural injection, alginate-based hydrogels form in situ, and ATP within the TME competitively binds to ATP-specific aptamers, triggering the release of immunoadjuvants and chemotherapeutic agents. This strategy enables tumour cell eradication while simultaneously enhancing immune memory against tumour cells and suppressing distant tumour metastasis [77]. Within DDS, ATP-responsive hydrogels are frequently integrated with other tumour treatment modalities to further enhance overall antitumour efficacy.

### 3.4. Redox-Responsive Hydrogels

Redox homeostasis refers to the dynamic balance between the generation and elimination of reactive oxygen species (ROS) [78]. Elevated oxidative stress is primarily associated with excessive production of oxidative species, such as singlet oxygen, hydroxyl radicals, and peroxides [79]. A reduced antioxidant capacity is mainly attributed to decreased GSH levels within the TME [80]. Compared with normal cells, tumour cells often overproduce intracellular GSH or ROS to maintain a strongly reductive environment or to induce heightened oxidative stress [81]. Consequently, the disruption of redox balance has emerged as a promising strategy for cancer therapy. Among these approaches, oxidation-responsive hydrogels utilise ROS to induce hydrogel degradation and subsequent drug release, while GSH-responsive systems incorporating disulfide bonds (–S–S–) trigger drug release in response to elevated intracellular GSH concentrations [82]. Notably, excessive GSH and ROS production may occur across different tumour types, within distinct regions of the same tumour, or at various stages of tumour progression [81]. Redox potential is a critical biological parameter reflecting the equilibrium of redox reactions and frequently fluctuates under pathological conditions, such as cancer, inflammation, and hypoxia [83]. Similarly to intracellular pH gradients, redox potential is regulated by elevated cytoplasmic and organelle GSH concentrations, creating opportunities for triggered intracellular drug delivery [84]. Redox-responsive nanocomposite hydrogels are designed to exploit these variations, typically incorporating reductively cleavable linkers (e.g., disulfides, diselenides, or thiol-maleimide bonds), ROS-sensitive moieties (e.g., diselenides, phenylboronic esters, thioketals, or thioethers), or metallic nanocomponents [85,86,87]. The pronounced disparity in redox potential between tumour and normal tissues offers a valuable opportunity for the development of intracellular DDS capable of regulating drug release, particularly within tumour sites [88]. Accordingly, GSH-/ROS-responsive targeted drug release has become an attractive strategy for the development of antitumour therapeutics.

In a landmark study, redox-responsive nanocomposite hydrogels were developed by exploiting the reversible Michael-type addition reaction between maleimide-functionalised liposomes and thiol-modified four-arm PEG polymers [89]. Stimulus-responsive peptide hydrogels have also attracted considerable attention as promising drug delivery platforms owing to their excellent solubility, superior biocompatibility, ease of modification, and capacity for redox-triggered drug release [88]. Furthermore, Mei’s team developed a redox-responsive Pep-CS-LND hydrogel capable of simultaneously targeting mitochondrial disulfide bonds and coupling KLAK to achieve selective drug release in cancer cells [88]. To improve drug loading capacity and sustain drug release, Zhu’s group designed a pH/redox dual-responsive peptide hydrogel that responds to the TME. PTX-loaded peptide hydrogels were injected intratumourally and continuously released antitumour agents under TME stimulation, thereby achieving optimal therapeutic efficacy while reducing toxicity and adverse effects [90]. Moreover, smart hydrogels with sol–gel transition behaviour and in situ crosslinking capabilities can be formulated as highly efficient injectable DDS [91]. Zhang’s team reported a novel multi-responsive injectable polyester hydrogel ((mPEG-PCL-Se)_2_); the degradation and drug release rates of which are regulated by H_2_O_2_ or GSH concentrations [92]. At physiological temperature, this hydrogel undergoes a sol–gel transition to form a semi-solid structure with excellent encapsulation efficiency and high drug loading capacity [92]. Overall, redox-triggered degradable hydrogels demonstrate substantial potential for controlled drug delivery applications.

#### 3.4.1. ROS-Responsive Hydrogels

ROS are widely present in various solid tumours and represent one of the distinctive biomarkers that differentiate cancer cells from normal cells. In oncology, ROS exert a dual regulatory role. At low concentrations, ROS promote tumour proliferation, invasion, and drug resistance through redox signalling pathways. In contrast, when intracellular ROS levels exceed the antioxidant capacity of cells, they induce lipid peroxidation, mitochondrial membrane disruption, and DNA fragmentation, thereby rapidly initiating ferroptosis, cell death, and immunogenic cell death [93]. Owing to the distinct redox states of cancer and normal cells, and because ROS levels in tumour cells are close to their oxidative tolerance threshold, tumour cells are more susceptible to oxidative stress than normal cells, particularly when exogenous stimuli further exacerbate oxidative stress [93]. In addition, the TME is characterised by mitochondrial electron leakage and elevated expression of NADPH oxidase, resulting in hydrogen peroxide (H_2_O_2_) concentrations that are 5–10 times higher than those in normal tissues [94]. Under these H_2_O_2_-enriched conditions, peroxidases (PODs) can catalyse substrate-induced ROS generation, thereby further enhancing intratumoural oxidative stress [93]. However, as the TME is typically hypoxic, ROS-responsive hydrogels can be rationally designed to exploit hypoxic conditions and construct corresponding nanohydrogel DDS. In this context, therapeutic agents are released in proximity to tumour sites to promote tumour cell death, with nanohydrogels enabling ordered drug release according to local oxygen availability.

During solid tumour growth, the extensive consumption of local blood supply to meet the energy demands of rapid proliferation results in sustained or intermittent hypoxia and nutrient deprivation [95]. Recurrent hypoxia–reoxygenation cycles promote elevated intracellular ROS levels, while mild oxidative stress further activates antioxidant enzymes and related reductive pathways, including the increased synthesis of reduced GSH. L-methionine is an important endogenous antioxidant [96] that can be oxidised by ROS to methionine sulfoxide, making it an ideal candidate for the synthesis of ROS-responsive polymers. Li’s group developed a methoxy polyethylene glycol-block-poly (L-methionine) (mPEG-b-PMet)-based ROS-responsive thermosensitive hydrogel. When anticancer drug-loaded hydrogels (Dox/R848/aPD-1@Gel) were injected into C57BL/6 mice, the formulation significantly inhibited tumour growth while continuously releasing encapsulated anticancer agents at tumour sites, thereby enhancing antitumour efficacy [97]. Moreover, local administration of these hydrogels was associated with low systemic cytotoxicity and good biodegradability. Zou’s team reported a DHcelPBG hydrogel that functions as an ROS-responsive self-release delivery platform. In a TME enriched with excessive ROS, the DHcelPBG hydrogel promoted the enhanced killing of 4T1 tumour cells [98]. Furthermore, this hydrogel was shown to modulate the PI3K/Akt signalling pathway, thereby accelerating apoptosis of 4T1 cells and effectively improving antitumour efficacy. However, uncertainties related to drug loading capacity, release sequence, and spatial distribution may limit the performance of composite hydrogels. To address these challenges, Li’s group developed a composite system consisting of thermosensitive hydrogels and ROS-responsive nanogels tailored to the TME, enabling precise sequential drug release to enhance molecular targeted therapy and amplify immune activation [99].

Beyond applications in tumour therapy, ROS-responsive hydrogels have also been explored in the treatment of neurological disorders. For example, in neurodegenerative diseases, Hu’s team developed an ROS-responsive injectable hydrogel capable of sensitively detecting oxidative stress and releasing GSH in a controlled manner, thereby modulating inflammation and restoring impaired immune microenvironments to promote tissue repair [100].

#### 3.4.2. GSH-Responsive Hydrogels

GSH is a critical antioxidant that plays essential roles in maintaining cellular redox homeostasis, protecting cells from oxidative damage, and supporting tumour growth and metastasis [101]. Under sustained oxidative stress, ROS can induce redox adaptation, leading to the upregulation of GSH and other antioxidant molecules [102]. Depletion of GSH increases the susceptibility of cancer cells to oxidative stress, thereby enhancing the cytotoxic effects of therapeutic agents [103]. Compared with normal tissues, the TME exhibits significantly higher GSH concentrations (approximately 10 mmol/L) [104]. On the basis of these differences between tumours and normal tissues [105], the development of GSH-responsive hydrogels as DDS for cancer therapy holds considerable promise for improving therapeutic efficacy and enhancing targeted drug release. As disulfide bonds are sensitive to GSH, they are commonly incorporated into nanohydrogels for DDS in cancer treatment [102]. Liu’s group prepared a novel polythioctic acid–polyethylene glycol (PEG-PTA) hydrogel via a two-step reaction. The disulfide bonds within this hydrogel interact with thiols from abundant GSH in the TME, leading to hydrogel degradation and subsequent drug release. In vitro and cellular studies demonstrated that the hydrogel degraded and released drugs only in the presence of GSH, indicating that injectable GSH-responsive hydrogels are promising DDS for cancer therapy [106]. Similarly, Mei’s group synthesised self-assembling Pep-CS-LND hydrogels that cleave disulfide bonds and release LND–KLAK conjugates under high-GSH conditions. Both in vitro and in vivo experiments showed extensive mitochondrial accumulation of the released agents and effective induction of tumour cell death, highlighting the substantial potential of Pep-CS-LND hydrogels for drug delivery and antitumour applications [88].

Enhancing drug accumulation at tumour sites and improving the bioavailability of delivered therapeutics are key strategies for increasing chemotherapy efficacy while reducing systemic side effects [107]. Injectable hydrogels represent promising localised DDS owing to their high water content and tissue-like softness, allowing for direct intratumoural administration within the TME for controlled drug release. Li’s team reported an injectable hydrogel system for the local delivery of tumour-targeted nanomicelles with GSH-responsive drug release, which significantly improved antitumour efficacy [107]. Ouyang’s group developed novel GSH-responsive nanohydrogels via active ester reactions between chitosan (CS, containing –NH_2_ groups) and N-hydroxysuccinimide (NHS)-containing crosslinkers. Following incubation in 10 mM GSH, more than 80% of DOX was released from the nanohydrogels. Moreover, under high-GSH conditions, DOX-loaded nanohydrogels exhibited pronounced antitumour activity against A549 cells [28]. Beyond GSH-responsive hydrogel DDS, alternative strategies aim to deplete GSH within the TME to suppress tumour growth. Zhang’s group constructed CS-BA/PVA-Cu^2+^-CDDP hydrogels that selectively respond to and degrade under simulated acidic TME conditions. Cu^2+^ ions released from the hydrogel deplete intracellular GSH and are reduced to Cu^+^. Importantly, GSH depletion reduces CDDP-GSH binding, thereby enabling rapid intracellular release of cisplatin (CDDP) in tumour cells and significantly enhancing its antitumour efficacy [108]. Similarly, Ning’s group developed an injectable thermosensitive hydrogel containing hollow copper sulfide nanoparticles and β-lapachone (Lap) (CLH). This hydrogel releases Cu^2+^ to deplete overexpressed GSH in the TME, while the generated Cu^+^ further exploits TME characteristics to initiate nanocatalytic reactions that produce highly toxic hydroxyl radicals, thereby promoting tumour cell death [109]. In addition, GSH levels can be reduced by disrupting glutamine metabolism using glutaminase inhibitors, such as BPTES. For example, a thermosensitive hydrogel incorporating FeSAZ (single-atom enzymes) and BPTES was developed; under infrared laser irradiation, the hydrogel releases FeSAZ and BPTES into tumour cells, where BPTES reduces intracellular GSH and markedly inhibits tumour cell growth [110].

### 3.5. Enzyme-Responsive Hydrogels

Many biologically important enzymes exhibit marked differences in expression and activity between healthy and diseased states [111]. For example, hyaluronidase is often highly expressed in tumour cells. Consequently, the design of ERHs has attracted considerable interest for biomedical applications such as targeted controlled drug release and tumour-specific drug delivery [112]. Enzyme responsiveness is typically introduced into hydrogels through enzyme-mediated crosslinking or the incorporation of enzyme-cleavable moieties [113], enabling on-demand drug release in response to specific enzymatic reactions. For effective function, ERHs generally require three key elements: (1) appropriate enzyme substrates (e.g., HA for hyaluronidase); (2) specific interaction between the substrate and the target enzyme in tumour cells; and (3) after enzymatic reaction, the hydrogel undergoes biodegradation or structural alteration, thereby releasing the loaded drug.

**Figure 4:**
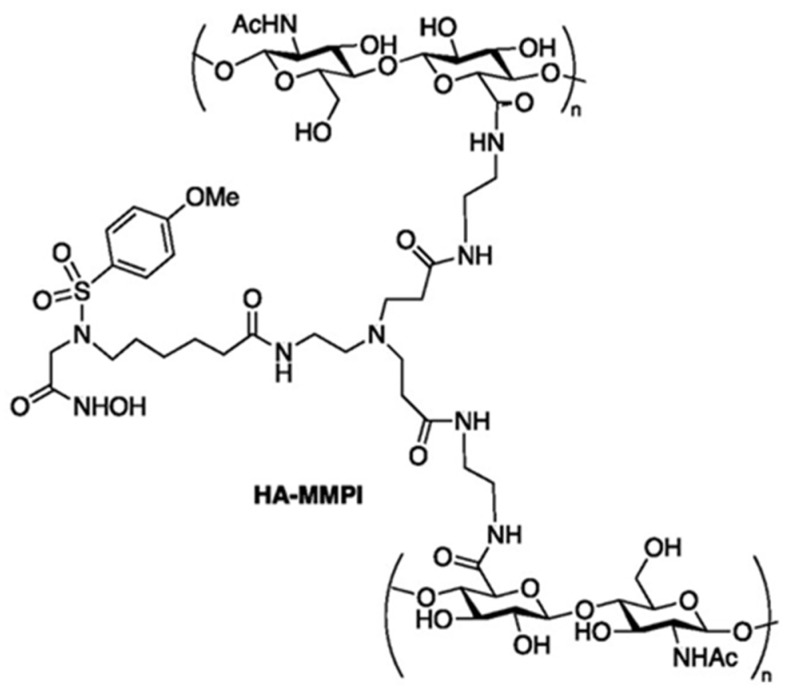
Schematic structure of crosslinked HA-MMPI [116].

Chemotherapy remains a cornerstone of cancer treatment, and to improve drug selectivity and reduce systemic toxicity, it is frequently combined with nanotechnology-based approaches. The integration of chemotherapeutic agents with nanohydrogel carriers that respond to the TME can enhance drug selectivity and tumour targeting [114]. Wang’s group developed an enzyme-responsive metallopeptide hydrogel (H_2_Yp–Pd) that enables the selective activation of tumour-targeted prodrug systems. In osteosarcoma cells overexpressing alkaline phosphatase, H_2_Yp–Pd selectively accumulates and, through palladium-mediated activation, promotes DOX release, resulting in pronounced cytotoxicity towards tumour cells while preserving normal cell viability [114]. Hyaluronidase is commonly overexpressed in tumour tissues, and this feature has been exploited in the design of DOX-loaded HA hydrogels. In microenvironments with high hyaluronidase activity, these hydrogels undergo specific enzymatic degradation, leading to the cleavage of the hydrogel network and sustained drug release. The released drugs are subsequently internalised by tumour cells and transported to the nucleus, achieving prolonged anticancer effects [115]. Furthermore, when HA is crosslinked with branched matrix metalloproteinase (MMP) inhibitors (MMPIs), a bioresponsive hydrogel can be constructed (Figure 4). Following surgery, this hydrogel enables in situ selective inhibition of MMP-2 (gelatinase A) within glioblastoma microenvironments, thereby reducing tumour cell migration [116]. Liu’s group demonstrated that hyaluronidase (HAase) can specifically degrade HA, facilitating enhanced drug penetration and cellular uptake of emodin. In vitro studies showed the inhibition of 4T1 cell proliferation and migration, while in vivo experiments revealed rapid gelation, sustained drug release, and strong antitumour effects in tumour-bearing mouse models, with a tumour growth inhibition rate of 73.23% [117]. In addition, HA-based hydrogels have been utilised to respond to the presence of HAase in urine for bladder cancer detection and subsequent evaluation of treatment efficacy [118].

Beyond their application in cancer drug delivery, ERHs have been extensively explored for therapeutic delivery in other diseases, tissue regeneration, the modulation of inflammatory responses, and the simulation of biological enzyme activities. Maki’s group reported a transparent supramolecular hydrogel formed from amphiphilic urea derivatives bearing hydrophilic lactose moieties. The lactose segments undergo enzymatic hydrolysis, and the introduction of β-galactosidase (β-Gal) induces a gel–sol phase transition, thereby triggering the controlled release of drugs encapsulated within the hydrogel [119]. Li’s group developed an alkaline phosphatase-responsive, self-administrable dexamethasone sodium phosphate hydrogel via ion-crosslinking strategies, which enabled the enzyme-triggered release of adalimumab for the treatment of autoimmune uveitis [120]. In addition to drug delivery, ERHs can be engineered to mimic endogenous antioxidant enzyme cascades, such as those of superoxide dismutase (SOD) and catalase (CAT), thereby efficiently scavenging ROS [121]. Agarwal’s team reported a self-assembling metallohydrogel with peroxidase-like activity that, under physiological conditions, catalytically decomposes H_2_O_2_ into H_2_O and O_2_, effectively alleviating cellular oxidative stress caused by excessive H_2_O_2_ accumulation [122]. Although enzymatic reactions are generally irreversible, recent studies have demonstrated that ERHs can exhibit remarkable self-healing capabilities through the formation of reversible covalent bonds [123]. Gao et al. successfully constructed an enzyme-responsive self-healing hydrogel by exploiting reversible covalent interactions between glutaraldehyde and lysine residues present in glucose oxidase, CAT, and bovine serum albumin [123]. This enzyme-regulated self-healing mechanism enables complete restoration of hydrogel structure and mechanical properties after damage, effectively overcoming the problem of irreversible functional loss commonly observed in conventional hydrogels. Notably, this enzyme-regulated self-healing hydrogel also exhibits pronounced antimicrobial activity, which provides broad-spectrum antibacterial efficacy against both Gram-negative bacteria (e.g., Escherichia coli) and Gram-positive bacteria (e.g., Staphylococcus aureus) [124]. Owing to their excellent biocompatibility and antimicrobial performance, these enzyme-assisted self-healing hydrogels are well-suited for use as wound dressings in skin repair.

## 4. Exogenous Stimuli

### 4.1. Photo-Responsive Hydrogels

Light represents a highly versatile external stimulus that enables precise spatiotemporal regulation of material behaviour and drug release [125]. Compared with endogenous stimuli (e.g., pH, temperature, proteases, and ATP) in biological systems, light affords superior control over the timing, location, and intensity of therapeutic payload release. Consequently, photo-responsive hydrogels capable of undergoing structural transformation or degradation upon light irradiation have attracted substantial attention. These hydrogels typically exhibit excellent stability under physiological conditions while enabling on-demand responsiveness under defined light exposure [125]. In ultraviolet (UV)-responsive hydrogel systems, a common design strategy involves replacing the aromatic capping groups essential for supramolecular self-assembly with photocleavable analogues. Upon brief UV irradiation, these capping groups are cleaved, yielding peptide fragments with enhanced hydrophilicity. This process disrupts π–π stacking interactions within the hydrogel network, leading to nanostructural disassembly and ultimately inducing gel–sol or gel–solution transitions [126]. In practical applications, photo-responsive hydrogel-based DDS are frequently integrated with photochemotherapy, PDT, or photothermal therapy (PTT) to achieve synergistic therapeutic outcomes.

Photochemical reactions relevant to hydrogel design mainly include photooxidation, photocleavage, and photopolymerization. Among these, photocleavage-triggered drug release is the most widely employed strategy. Ortho-nitrobenzyl derivatives are among the most commonly used photocleavable linkers and can be readily incorporated into hydrogel matrices to impart photo-responsiveness. Upon exposure to UV or high-energy visible light, cleavage of the ester C-O bonds in ortho-nitrobenzyl groups occurs, inducing changes in hydrogel network architecture and facilitating controlled drug release [127]. By embedding photocleavable linkers within polymer backbones or crosslinking points, light-triggered degradation of hydrogels can be precisely regulated. Beyond drug delivery, photochemical approaches have emerged as powerful tools in tissue engineering and mechanobiology for dynamically tuning hydrogel microenvironments. The incorporation of photosensitive functional groups into polymer networks enables the spatiotemporal modulation of mechanical properties and degradation behaviour through controlled light exposure [128,129]. Upon irradiation at specific wavelengths, photodegradable hydrogels lose their load-bearing capacity, exhibiting high dynamic tunability—an attribute particularly advantageous for controlled drug delivery. For example, Zhao’s group developed a photodegradable injectable self-healing hydrogel containing photolabile ortho-nitrobenzyl ester moieties. Under UV irradiation, hydrophobic segments were converted into hydrophilic domains, significantly accelerating DOX release and enhancing tumour cell apoptosis [130].

The therapeutic mechanism of PDT relies on photosensitisers (PSs) that, upon light activation, transfer energy to molecular oxygen to generate highly cytotoxic singlet oxygen via type II photochemical reactions, thereby selectively damaging tumour tissues. However, the short diffusion distance and limited lifetime of singlet oxygen restrict PDT efficacy. Encapsulation of PSs within hydrogel matrices enhances local retention and accumulation at tumour sites, enabling “dose reduction with efficacy enhancement.” Manzar’s group developed an Fmoc-FF/polylysine (PLL) hydrogel incorporating chlorin e6 (Ce6) as a PS. In vivo studies demonstrated that a single injection combined with multiple light irradiations effectively suppressed tumour growth [131]. Similarly, Zheng and colleagues reported an in situ injectable thermosensitive hydrogel loaded with P-TTPy PSs, which significantly prolonged PS retention and enabled sustained PDT for lung cancer treatment [132]. Moreover, by integrating persistent luminescent materials and immunoadjuvants (R837) into calcium alginate hydrogels, persistently luminescent hydrogels were constructed. Acting as internal light sources, these systems continuously activate PSs and achieve prolonged PDT efficacy [133].

In contrast to PDT, which depends on ROS, PTT utilises photothermal conversion agents to transform absorbed light energy into heat, inducing localised hyperthermia and direct tumour ablation [134]. This therapeutic modality is not limited by hypoxic tumour environments and is effective against drug-resistant cancer cells. The immobilisation of photothermal agents (such as gold nanorods, polydopamine nanoparticles, or two-dimensional nanomaterials) within hydrogel networks enables in situ retention following injection or implantation, reducing systemic toxicity while ensuring stable photothermal performance. Chen’s group developed photosensitiser-loaded hydrogel liposomes for PTT, which efficiently delivered PSs to both subcutaneous tumours and deep metastatic lesions, resulting in marked tumour growth inhibition after laser irradiation [135]. Hyperthermia-based cancer therapy exploits the thermal vulnerability of tumour cells. Elevating local tissue temperatures to 43–46 °C reduces cancer cell viability and enhances sensitivity to chemotherapy and radiotherapy [136]. Notably, mild hyperthermia can deplete intracellular ATP and suppress heat-shock protein expression, thereby overcoming tumour thermotolerance and enabling effective tumour ablation at relatively low temperatures (43 °C) [137]. Beyond oncological applications, photo-responsive hydrogels also show promise in antibacterial therapy, where light-triggered state transitions enable localised and controlled drug release at wound sites.

To overcome the inherent limitations of single-modality PDT or PTT, combinatorial therapeutic strategies have been actively explored. Sun’s group developed a collagen–gold composite hydrogel enabling synergistic PDT/PTT, achieving tumour eradication rates of up to 80% in vivo [138]. Similarly, Zhang’s group engineered a dopamine-modified hydrogel loaded with Ce6, enabling simultaneous PDT and PTT under near-infrared irradiation. In this system, Ce6 generates cytotoxic ROS for PDT, while the polydopamine component rapidly converts light into heat for PTT, effectively eliminating primary tumours and significantly suppressing recurrence [139]. Overall, such synergistic photo-responsive hydrogel platforms markedly enhance therapeutic efficacy while addressing the intrinsic shortcomings of single-modality treatments.

### 4.2. Magnetothermal-Responsive Hydrogels

Magnetic hyperthermia therapy (MHT) has emerged as a minimally invasive approach in biomedical applications, utilising the unique properties of magnetic nanoparticles (MNPs) to generate localised heat under external alternating magnetic fields (AMFs), thereby inducing tumour cell ablation [140]. Directing MNPs to target tumour tissues and applying AMFs enhances anticancer efficacy by producing controlled localised heating. Compared with light-based modalities, AMFs penetrate tissues more deeply, enabling treatment of tumours at greater depths [141].

Unlike PTT, magnetic nanomaterial-mediated thermotherapy converts electromagnetic energy directly into heat, allowing for tumour ablation without depth limitations under AMFs [142]. Qian and colleagues developed an injectable ferrimagnetic silk fibroin hydrogel. Following intratumoral injection, exposure to AMFs induced effective localised hyperthermia, achieving tumour cell ablation in vivo [143]. Xie’s group incorporated commercial magnetic metal or metal oxide powders (CMMPs) into alginate-Ca^2+^ hydrogels (ALG-Ca^2+^), generating an injectable ALG-Ca^2+^-CMMP system. After injection around tumour sites, the CMMPs were retained, improving targeting precision and reducing collateral effects. Under AMF irradiation, this system produced localised heat for tumour ablation [144].

Magnetoresponsive hydrogels not only facilitate hyperthermia but also function as DDS. These hydrogels exploit magnetically induced thermal transitions to self-regulate temperature, while simultaneously releasing chemotherapeutic agents (such as adriamycin hydrochloride and DOX) over extended periods (>120 h), thereby enhancing DOX utilisation [145]. Yin’s team developed a core–shell hydrogel microsphere system integrating sequential drug release with magnetothermal therapy. The system initially releases inhibitors to disrupt drug-resistance pathways, sensitising tumour cells, followed by cisplatin release for sustained cytotoxicity. MNPs generate temperatures of 42–46 °C under AMFs, inducing apoptosis and potentiating cisplatin efficacy [146].

### 4.3. Ultrasound-Responsive Hydrogels

Ultrasound has emerged as an attractive external stimulus for smart hydrogel systems due to its non-invasive nature, high tissue penetration, and precise spatiotemporal control [147]. Ultrasound-responsive hydrogels undergo direct structural changes under ultrasonic stimulation, enabling targeted and controlled intratumoral drug release [148]. Both mechanical (nonthermal) and thermal effects induced by ultrasound serve as mechanisms for drug release [149]. Tumour-focused ultrasound generates thermal effects that increase vascular permeability and blood flow, improving drug access to tumour tissues and accelerating hydrogel-mediated drug release [150]. After the cessation of ultrasound, thermal reversibility allows hydrogels to return to their original gel state via sol–gel transitions [151].

Ultrasound can also mediate controlled drug release from composite hydrogels. Wu and colleagues developed an N-isopropylacrylamide-based hydrogel capable of ultrasound-triggered release of macromolecules such as bovine serum albumin and dextran. Release rates increased with elevated hydrogel temperature, demonstrating thermosensitive behaviour [152]. Liu’s group designed an ultrasound-responsive Pluronic P105/F127 nanogel to overcome multidrug resistance in cancer. In vitro studies showed that ultrasound enhanced DOX release, facilitating greater cellular uptake and significantly increasing cytotoxicity in human breast cancer cells after just three minutes of exposure [153]. Ultrasound-mediated sonodynamic therapy (SDT) represents another application, mechanistically similar to PDT [154]. SDT activates drugs to generate ROS in tumour cells, thereby triggering immune responses that inhibit tumour progression and metastasis. Furthermore, SDT induces oxidative stress, endoplasmic reticulum stress, DNA damage, and mitochondrial membrane potential loss, indicating precise control over drug activity in resistant tumour cells [155]. Composite hydrogels incorporating sonosensitisers enable simultaneous ultrasound-responsive drug release and ROS generation, exerting synergistic anticancer effects.

## 5. Multiple Stimulus Sources

TME is often complex and highly specific. Single-stimulus-responsive hydrogels in drug delivery frequently encounter limitations, including insufficient targeting precision and restricted response efficiency. Consequently, developing hydrogels capable of synergistically responding to multiple stimuli (such as low pH, specific enzymes, elevated GSH concentrations, and ROS) has become a critical strategy for achieving precise and efficient tumour therapy (Table 2) [156].

### 5.1. pH–Redox Dual Stimulation

pH–Redox dual-responsive hydrogels represent a novel class of targeted biomaterials, capable of precisely responding to the TME and achieving effective drug delivery with in situ-controlled release. These hydrogels exploit the synergistic response to tumour-specific redox potential differences and acidic microenvironments, significantly enhancing therapeutic efficacy while minimising toxic side effects on normal tissues [167]. At the redox-responsive level, the incorporation of disulfide bond-functionalised crosslinkers enables hydrogel degradation in response to redox potential. Disulfide bonds can be orthogonally cleaved via thiol–disulfide exchange reactions with reducing agents, such as dithiothreitol or GSH. This results in hydrogel breakdown into nontoxic, water-soluble products [168]. At the pH-responsive level, these hydrogels contain functional groups sensitive to acidic or basic environments, allowing them to swell or contract in response to local pH variations. This facilitates selective drug release under the acidic conditions characteristic of the TME [56]. The design and characterisation of such hydrogels typically focus on verifying pH-sensitivity, redox-specific degradation, and controlled drug release. For instance, Yang’s team developed an injectable pH–redox dual-responsive hydrogel loaded with combretastatin-A4 phosphate (CA4P) and DOX. Sequential local delivery of CA4P and DOX induced tumour vascular atrophy and apoptosis, demonstrating synergistic therapeutic effects [169].

Research indicates that the TME can simultaneously exhibit acidic pH and elevated ROS levels [170,171,172]. Boronic ester bond-based hydrogels are highly sensitive to ROS, as the boron atoms possess empty p orbitals that readily react with H_2_O_2_. This reaction, akin to a Baeyer–Villiger oxidative rearrangement, triggers hydrolysis and cleavage of the hydrogel linkages [173]. Similarly, Schiff base bond-based hydrogels undergo hydrolytic cleavage under acidic conditions, enabling pH-responsive degradation [174]. Yi and colleagues developed an injectable ROS/pH-responsive hydrogel via Schiff base crosslinking of thioketone-based ROS-cleavable linkers with aldehyde-functionalised HA. This hydrogel degraded under oxidative and acidic conditions, enabling sequential drug release in breast tumour models [175]. Dual-responsive supramolecular hydrogels accelerate drug release upon exposure to H_2_O_2_ or low pH, demonstrating efficient dual-stimulus-controlled delivery [176]. For example, hydrazone bond-containing PCMS@PAC/α-CD Gel released cinnamaldehyde and amplified H_2_O_2_ levels under acidic conditions, exhibiting high cytotoxicity and effective tumour inhibition in vitro and in vivo [177].

TMEs commonly display both acidic microenvironments and elevated intracellular GSH concentrations [178]. Designing DDSs that respond to both stimuli is, therefore, a promising research direction. Incorporating reductively cleavable bonds (such as disulfide, diselenide, or selenium–sulfur bonds) enables hydrogels to respond to high GSH levels within tumour cells. In such conditions, these bonds are specifically cleaved, triggering rapid redox responses, hydrogel degradation, and precise drug release [179,180]. Wang and colleagues reported a PEG-DTP/ADA hydrogel incorporating pH-sensitive acylhydrazone bonds and redox-sensitive disulfide bonds, allowing for hydrogel degradation to be modulated by pH or reducing agent concentrations [181]. Similarly, Hu’s group developed a Salecan-grafted poly (acrylic acid-co-hydroxyethyl methacrylate) hydrogel (Salecan-g-SS-poly (IA-co-HEMA)) with dual responsiveness: carboxylic acid groups conferred pH sensitivity, controlling swelling behaviour according to environmental pH and Salecan content, while disulfide bonds provided selective degradation in reductive tumour environments. This combination of stimuli-responsive swelling and cleavage underscores the hydrogel’s potential as a smart anticancer drug delivery platform [168].

In summary, pH–redox dual-responsive hydrogels precisely regulate drug loading and release through dynamic bond formation and cleavage, such as disulfide bonds [181,182]. Compared with single-stimulus DDS, these multi-responsive systems synergistically respond to both acidic TMEs and intracellular redox potential differences, achieving more accurate tumour-targeted drug delivery.

### 5.2. pH–Temperature Dual Stimulation

Smart hydrogel design strategies for tumour drug delivery are evolving from single-stimulus responses towards multi-stimulus synergistic systems [183]. Temperature-responsive hydrogels exemplify this approach, utilising temperature-triggered sol–gel phase transitions to transform from injectable liquids into in situ gels [184]. This property enables sustained drug release at tumour sites, maintaining effective local concentrations and ensuring therapeutic efficacy. However, drug release in purely temperature-responsive systems primarily relies on physical diffusion and lacks the active recognition of tumour-specific pathological features. To overcome this limitation, pH–temperature dual-responsive hydrogels have been developed [185]. pH-responsive hydrogels specifically react to the acidic conditions of the TME [186], achieving “on-demand” drug release through chemical bond cleavage, thereby enhancing local targeting [187]. Consequently, multi-responsive hydrogels integrating temperature and pH responses have become a major research focus. Lin and colleagues constructed pH–temperature-responsive monomethoxy polyethylene glycol (mPEG)-polypeptide hydrogels. When the pH increased from 6.5 to 7.0, mPEG-polypeptide aqueous solutions underwent α-helix to β-sheet transitions, leading to markedly reduced gel concentrations and enhanced drug release at neutral pH. Furthermore, local temperature increases induced sol–gel phase transitions. Subcutaneous degradation studies in mice revealed complete hydrogel degradation within three weeks, demonstrating good biocompatibility [188]. Similarly, Jommanee et al. investigated an injectable hydrogel exhibiting both pH and temperature dual-responsiveness. Its sol–gel phase transition behaviour was uniquely tunable: the gelation temperature decreased with increasing pH. In vitro cytotoxicity assays confirmed excellent biocompatibility, establishing its potential for biomedical applications [189].

### 5.3. Redox/Ultrasound Dual Stimulation

Ultrasound, owing to its safety, non-invasiveness, and high spatiotemporal controllability, has emerged as an ideal tool for constructing exogenous stimulus-responsive DDS [190]. In contrast, the high-level redox stress characteristic of tumour cells, such as elevated GSH concentrations, represents a potent endogenous trigger [191]. Combining exogenous ultrasound stimulation with tumour-endogenous redox triggers enables a dual- or multi-level responsive DDS, achieving synergistically enhanced, precise drug release. This strategy overcomes the limitations of single-stimulus systems, such as insufficient sensitivity or restricted targeting, while significantly improving drug enrichment and release efficiency at tumour sites through an “internal–external pincer” approach. Kumar and colleagues developed a redox–ultrasound dual-responsive nanogel system for precise drug release regulation. Thermosensitive PEIm–PNIPAMn–PEIm copolymers self-assemble into micelles in aqueous solution above their lower critical solution temperature. Subsequent crosslinking with disulfide bond-containing linkers forms PEI gel shells, producing spherical nanogels. The encapsulation of perfluorohexane (PFH) within the nanogel cores generates cavitation effects under ultrasound stimulation, facilitating rapid drug release. Concurrently, the PEI gel shells are specifically degraded by high GSH concentrations in the TME, providing redox-responsive release. These mechanisms collectively accelerate release rates, achieving near-instantaneous complete drug delivery. The study confirmed that dual-stimulus-responsive nanogels offer promising prospects for precisely controlled drug delivery [192].

Unlike single-stimulus systems, multi-stimulus-responsive hydrogels require the integration of multiple responsive functional groups within a single polymer network to enable sequential or concurrent drug release under different stimuli. Precise control over hydrogel properties (such as swelling behaviour, mechanical strength, and degradation rate) is critical, as these parameters are influenced by monomer selection, crosslinking density, and synthesis conditions. Distinct stimuli trigger specific release mechanisms: pH-sensitive bonds (e.g., Schiff bases) hydrolyse in acidic TMEs; disulfide bonds respond to elevated intracellular GSH levels; temperature-responsive groups induce sol–gel phase transitions at physiological temperatures; and enzyme-cleavable peptides enable tumour-specific release. Interactions among multiple stimuli may be synergistic or competitive. For example, acidic TMEs and high GSH levels can jointly accelerate bond cleavage, enhancing drug release. To mitigate these challenges, strategies include: orthogonal design, ensuring each stimulus operates independently; spatial compartmentalisation, physically segregating therapeutic agents to prevent interference while permitting independent control; engineered response thresholds, activating pH-sensitive groups only below pH 6.0 and temperature-sensitive groups at 40 °C to avoid premature release; dynamic covalent chemistry, enabling reversible stimulus-responsive bonds to maintain mechanical stability; and machine learning approaches, optimising interactions among multiple response mechanisms. In summary, the development of multi-stimulus-responsive hydrogels for precision tumour therapy necessitates integrated strategies that address synthetic complexity, precise control of release kinetics, and potential stimulus interactions. These considerations represent a key frontier in advancing smart DDS.

## 6. Conclusions and Perspectives

Stimuli-responsive hydrogels represent highly promising carriers for precision tumour drug delivery. In recent years, due to their unique physical and chemical properties and biocompatibility, they have made substantial progress in overcoming the limitations of traditional tumor therapies (i.e. surgery, chemotherapy, and radiotherapy), including poor targeting, significant side effects, and limited efficacy. Precise regulation of drug release mechanisms constitutes the foundation of these systems. Smart hydrogels primarily deliver drugs via two mechanisms: Fickian diffusion and chemically controlled release. Chemically controlled release relies on reversible or irreversible chemical bond formation and cleavage, effectively preventing burst release, enabling zero-order or on-demand release, and significantly enhancing drug utilisation efficiency. This mechanism predominates in responsive hydrogels. Biocompatibility, biodegradability, and non-toxicity remain essential attributes for all biomaterials intended for medical applications. Among endogenous stimuli, pH, temperature, ATP, ROS, GSH, and enzymes naturally exist within the human body; thus, internally stimulus-responsive hydrogels can achieve site-specific drug delivery. External stimuli, including light and electric fields, exhibit slower response times and are frequently combined with endogenous cues to achieve localised drug delivery and enhanced therapeutic efficacy.

Despite considerable advances in this research field, a majority of current research remains at the preclinical stage, including in vitro cellular studies and animal models, with only a few hydrogels progressing to early clinical trials. Limitations persist in biosafety evaluation, precise regulation of response sensitivity, and optimisation for large-scale manufacturing, creating substantial barriers to clinical translation. From a regulatory perspective, hydrogels are generally classified as “devices” under the U.S. Federal Food, Drug, and Cosmetic Act, requiring FDA 510(k) pre-market review, whereas hydrogels encapsulating drugs are considered “combination products” and face longer approval timelines, constraining commercialisation. Nonetheless, clinical translation remains challenged by large-scale production difficulties, batch consistency, variations in response precision, and complex regulatory classifications. Addressing these issues requires adherence to Good Manufacturing Practices (GMP), robust process validation, real-time monitoring, automated and continuous manufacturing processes, and enhanced collaboration among academia, industry, and regulatory bodies. Long-term stability and in vivo degradation characteristics critically influence clinical translation potential. Sustained stability allows hydrogels to provide prolonged drug release via implantation, effectively inhibiting tumour growth. Degradation predominantly occurs through hydrolysis and enzymatic pathways, with in vivo behaviour affected by implantation site, species differences, and local microenvironmental factors, such as pH, temperature, and enzyme concentration. Stimuli-responsive hydrogels can exploit TME characteristics to modulate degradation: for instance, pH-responsive hydrogels leverage acidic TME to trigger drug release, while ERHs (e.g., hyaluronan-based) degrade effectively in enzyme-rich tumour regions.

Future research directions for stimuli-responsive hydrogels in precision tumour therapy should target current limitations and clinical demands. These include the following: enhancing nano-responsiveness to improve drug delivery precision by introducing highly efficient responsive groups and lowering activation thresholds to maximise TME specificity while minimising normal tissue damage; expanding biocompatible and biodegradable material systems, exploring novel hydrogels (DNA-based, supramolecular, and biomimetic) with advanced functionalities, such as self-healing, chemotaxis, and immunoregulatory activity; advancing surface nano-modification techniques to reduce immunogenicity, improve circulation stability, and enhance tumour penetration; and developing personalised nanomedicine strategies by integrating single-cell sequencing and patient-specific tumour characterisation to optimise response types, drug-loading, and administration routes. Further directions include designing dynamic nano-regulation strategies to control release kinetics according to tumour type and growth stage, and integrating smart hydrogels with multimodal therapeutic technologies—including chemotherapy, immunotherapy, PDT, and gene editing. For example, multi-responsive hydrogels loaded with chemotherapy agents and immunoadjuvants can deliver drugs precisely while promoting immunogenic cell death and activating systemic antitumour immunity, thereby overcoming drug resistance and metastatic progression.

In summary, the principal advantages of stimuli-responsive hydrogels lie in their multi-stimulus responsiveness, achieved through degradable polymer selection (natural, synthetic, or composite), precise surface nano-modification, and scientifically engineered network structures. These hydrogels enhance tumour-targeted delivery and therapeutic efficacy while overcoming the limitations of single-response systems. Future efforts should focus on optimising material systems and nano-scaffold design, integrating multimodal therapeutic strategies into unified delivery platforms, addressing scale-up manufacturing and biosafety challenges, and advancing personalised clinical treatment regimens. Pursuing these directions will accelerate the translation of smart hydrogels from preclinical research to clinical application, providing implementable nanomedicine strategies to overcome tumour drug resistance and achieve personalised precision therapy, thereby realising their full potential in tumour precision medicine.

## Figures and Tables

**Table 2:** Multi-stimulus-responsive hydrogels.

NO.	Hydrogel	Response Type (Multiple Stimuli)	Mechanism	Tumour Type	Ref.
1	HA-NCs	PH/GSH	This hydrogel passively accumulates in tumour cells through enhanced EPR effects and cellular uptake, while responding to dual stimuli of GSH and low pH. High GSH levels and low pH within tumours trigger the rupture of hydrogel nanovesicles, promoting the release of the anticancer drug (DOX).	-	[157]
2	Nanohydrogel/MTX	pH/reduction response	pH- and reduction-responsive magnetic nanohydrogels loaded with the anticancer drug methotrexate (MTX) achieved a drug loading efficiency (LE) of 64 ± 2.7%. Drug release occurs upon stimulation by the TME.	-	[158]
3	FA-DOX-Ind-NP	pH/reduction response	This nanoparticle (FA-DOX-Ind-NP) exhibits dual pH/redox-responsive drug release characteristics, enabling simultaneous and controlled delivery of the chemotherapy drug DOX and the nonsteroidal anti-inflammatory drug indomethacin within the TME.	-	[159]
4	HA-BP·MOF@DOX hydrogel	pH/ATP	Based on the release kinetics observed in this study, low pH can induce DOX release, while ATP accelerates DOX release. Under low pH conditions and high ATP levels, the release duration of DOX within the TME is also prolonged.	CT-26 tumour-bearing mice	[160]
5	Polymeric hydrogel	pH/temperature	This hydrogel specifically responds to endogenous biological stimuli within tumour cells, such as acidic pH and temperature. In vitro experiments confirm that the hydrogel exhibits significant targeted release of DOX under acidic conditions and demonstrates excellent cancer cell killing efficacy.	Human breast cancer (MCF-7) and human cervical carcinoma cells (HeLa)	[161]
6	Stimuli-responsive copolymeric hydrogels (CH)	pH/temperature/reduction	This study developed a multifunctional hydrogel for achieving triple synergistic control of temperature, reduction, and pH dependent release of the anticancer drug 5-fluorouracil (5-FU). In vitro cytotoxicity assays demonstrated that the blank hydrogel (drug-free) exhibited excellent biocompatibility. In contrast, the drug-loaded hydrogel (CH) displayed significant cytotoxicity toward human hepatocellular carcinoma SMMC-7721 cells, with cell viability reduced to approximately 25% at concentrations as low as 1 mg mL^−1^.	Human hepatoma SMMC-7721 cells	[162]
7	Composite Hydrogel	pH/redox/magnetothermotherapy	This study designed and developed a novel magnetite pH/redox-responsive drug delivery system based on natural *Tragacanth gum* extract for solid tumour therapy. The results demonstrated that the developed DDS exhibits superior anticancer activity (approximately 24% enhancement) due to its “smart” sustained-release properties and synergistic effects with hyperthermia.	-	[163]
8	TiOx@CaO_2_aPD-L1-gels	pH/ROS	Due to the cleavage of the phenylboronic ester bond, the internal payload can be controlled to release in response to the weakly acidic pH and ROS in the TME. Among these, TiOx@CaO_2_ can serve as an efficient sonosensitiser or Fenton-like agent to generate high levels of ROS, thereby enhancing SDT or CDT and effectively inhibiting tumour cell growth.	-	[164]
9	MAA-co-NIPAM	pH/Temperature	The hydrogel modifies the pH and temperature sensitivity of CS by crosslinking with other groups/monomers to enhance CS’s performance as a drug carrier and ensure drug loading efficiency.		[165]
10	DNA–acrylamide-based hydrogel	ATP/pH	Experiments revealed that ATP- or pH-responsive microcapsules containing the anticancer drug DOX exhibit selective cytotoxicity toward MDA-MB-231 cancer cells.	MDA-MB-231 cancer cells	[166]

## Data Availability

The original contributions presented in this study are included in the article. Further inquiries can be directed to the corresponding author.

## Abbreviations

The following abbreviations are used in this manuscript:

GSH	Glutathione
H_2_O_2_	Higher hydrogen peroxide
ROS	Reactive oxygen species
DOX	Doxorubicin
DDSs	Drug delivery systems
CS	Chitosan
PEG	Polyethylene glycol
PTX	Paclitaxel
TME	Tumour microenvironment
SOD	Superoxide dismutase
CAT	Catalase
CDT	Chemodynamic therapy
PDT	Photodynamic therapy
PTT	Photothermal therapy
PSs	Photosensitisers
MHT	Magnetic hyperthermia therapy
MNPs	Magnetic nanoparticles
AMFs	Alternating magnetic fields
UV	Ultraviolet
SDT	Sonodynamic therapy
Ce6	Chlorin e6
HA	Hyaluronic acid
ERHs	Enzyme-responsive hydrogels

## References

[1] Gao F., Guo L., Lin W., Zhang X., Zhan Q., Cao P., Ju H., Zhang Y. (2025). Simply Designed and Universal DNA Nanohydrogel for Stimuli-Responsive NIR-II Fluorescence Imaging of Early-Stage Tumor. Anal. Chem..

[2] Hong L., Li W., Li Y., Yin S. (2023). Nanoparticle-based drug delivery systems targeting cancer cell surfaces. RSC Adv..

[3] Tang X., Li D., Gu Y., Zhao Y., Li A., Qi F., Liu J. (2022). Natural cell based biomimetic cellular transformers for targeted therapy of digestive system cancer. Theranostics.

[4] Xia W., Tao Z., Zhu B., Zhang W., Liu C., Chen S., Song M. (2021). Targeted Delivery of Drugs and Genes Using Polymer Nanocarriers for Cancer Therapy. Int. J. Mol. Sci..

[5] Xu H., Fei Y., Wang X., Jiao W., Jin Y. (2025). Advances in Hydrogel-Based Delivery of RNA Drugs for Antitumor Therapy. Gels.

[6] Le Y., Zhu S., Peng H., Wang Z. (2025). Unveiling the omics tapestry of B-acute lymphoblastic leukemia: Bridging genomics, metabolomics, and immunomics. Sci. Rep..

[7] Bejarano L., Jordāo M.J.C., Joyce J.A. (2021). Therapeutic Targeting of the Tumor Microenvironment. Cancer Discov..

[8] Qian C., Zhao G., Huo M., Su M., Hu X., Liu Q., Wang L. (2024). Tumor microenvironment-regulated drug delivery system combined with sonodynamic therapy for the synergistic treatment of breast cancer. RSC Adv..

[9] Vasile C., Pamfil D., Stoleru E., Baican M. (2020). New Developments in Medical Applications of Hybrid Hydrogels Containing Natural Polymers. Molecules.

[10] Yari-Ilkhchi A., Ebrahimi-Kalan A., Farhoudi M., Mahkam M. (2021). Design of graphenic nanocomposites containing chitosan and polyethylene glycol for spinal cord injury improvement. RSC Adv..

[11] Cao H., Duan L., Zhang Y., Cao J., Zhang K. (2021). Current hydrogel advances in physicochemical and biological response-driven biomedical application diversity. Signal Transduct. Target. Ther..

[12] Kong W.Q., Gao C.D., Hu S.F., Ren J.L., Zhao L.H., Sun R.C. (2017). Xylan-Modified-Based Hydrogels with Temperature/pH Dual Sensitivity and Controllable Drug Delivery Behavior. Materials.

[13] Li L., Zheng X., Pan C., Pan H., Guo Z., Liu B., Liu Y. (2021). A pH-sensitive and sustained-release oral drug delivery system: The synthesis, characterization, adsorption and release of the xanthan gum-graft-poly(acrylic acid)/GO-DCFP composite hydrogel. RSC Adv..

[14] Niewolik D., Bednarczyk-Cwynar B., Ruszkowski P., Kazek-Kęsik A., Dzido G., Jaszcz K. (2022). Biodegradable and Bioactive Carriers Based on Poly(betulin disuccinate-co-sebacic Acid) for Rifampicin Delivery. Pharmaceutics.

[15] Mohammadpanah F., Behrooz R., Pooyan M., Gholivand K., Roohzadeh R. (2025). Development of a phosphoaminopyrazine-loaded cellulose nanoparticle drug delivery system for targeted treatment of triple-negative breast cancer. Int. J. Biol. Macromol..

[16] Li X., Xu X., Xu M., Geng Z., Ji P., Liu Y. (2023). Hydrogel systems for targeted cancer therapy. Front. Bioeng. Biotechnol..

[17] Xiao C., Wang R., Fu R., Yu P., Guo J., Li G., Wang Z., Wang H., Nie J., Liu W. (2024). Piezo-enhanced near infrared photocatalytic nanoheterojunction integrated injectable biopolymer hydrogel for anti-osteosarcoma and osteogenesis combination therapy. Bioact. Mater..

[18] Rizwan M., Yahya R., Hassan A., Yar M., Azzahari A.D., Selvanathan V., Sonsudin F., Abouloula C.N. (2017). pH Sensitive Hydrogels in Drug Delivery: Brief History, Properties, Swelling, and Release Mechanism, Material Selection and Applications. Polymers.

[19] Mozafari R., Mohammadi M., Moradi S., Ghadermazi M. (2025). In situ synthesis of ultrafine Cu(ii) metal immobilized on pectin hydrogel, modified by a CoFe_2_O_4_/Pr-SO_3_H nanocomposite as a green catalyst for reduction of nitro compounds and synthesis of 1H-tetrazoles. RSC Adv..

[20] Hauptstein J., Böck T., Bartolf-Kopp M., Forster L., Stahlhut P., Nadernezhad A., Blahetek G., Zernecke-Madsen A., Detsch R., Jüngst T. (2020). Hyaluronic Acid-Based Bioink Composition Enabling 3D Bioprinting and Improving Quality of Deposited Cartilaginous Extracellular Matrix. Adv. Healthc. Mater..

[21] Micale N., Citarella A., Molonia M.S., Speciale A., Cimino F., Saija A., Cristani M. (2020). Hydrogels for the Delivery of Plant-Derived (Poly)Phenols. Molecules.

[22] Aswathy S.H., Narendrakumar U., Manjubala I. (2020). Commercial hydrogels for biomedical applications. Heliyon.

[23] Zhang Y., Wu B.M. (2023). Current Advances in Stimuli-Responsive Hydrogels as Smart Drug Delivery Carriers. Gels.

[24] Sun Y., Ren Z., Zhang Z., Yang K., Jin Y., Deng H., Liu Y., Wang J., Ji P., Liu P. (2025). Triple synergistic enhancement of breast cancer treatment via chemotherapy, chemodynamic therapy, and tumor starvation therapy driven by lipid-COF nanoparticles. Sci. Rep..

[25] Delgado-Pujol E.J., Martínez G., Casado-Jurado D., Vázquez J., León-Barberena J., Rodríguez-Lucena D., Torres Y., Alcudia A., Begines B. (2025). Hydrogels and Nanogels: Pioneering the Future of Advanced Drug Delivery Systems. Pharmaceutics.

[26] Zhang Q., Hu W., Guo M., Zhang X., Zhang Q., Peng F., Yan L., Hu Z., Tangthianchaichana J., Shen Y. (2024). MMP-2 Responsive Peptide Hydrogel-Based Nanoplatform for Multimodal Tumor Therapy. Int. J. Nanomed..

[27] Wang Y., Wu J., Chen M., Zhang J., Sun X., Zhou H., Gao Z. (2024). Application of near-infrared-activated and ATP-responsive trifunctional upconversion nano-jelly for in vivo tumor imaging and synergistic therapy. Biosens. Bioelectron..

[28] Ouyang C., Deng M., Tan X., Liu Z., Huang T., Yu S., Ge Z., Zhang Y., Ding Y., Chen H. (2024). Tailored design of NHS-SS-NHS cross-linked chitosan nano-hydrogels for enhanced anti-tumor efficacy by GSH-responsive drug release. Biomed. Mater..

[29] Wang J., He H., Cooper R.C., Gui Q., Yang H. (2019). Drug-Conjugated Dendrimer Hydrogel Enables Sustained Drug Release via a Self-Cleaving Mechanism. Mol. Pharm..

[30] Yang J., Qu J., Teng X., Zhu W., Xu Y., Yang Y., Qian X. (2023). Tumor Microenvironment-Responsive Hydrogel for Direct Extracellular ATP Imaging-Guided Surgical Resection with Clear Boundaries. Adv. Healthc. Mater..

[31] Kim M., Lee J.i., Choi J., Kim S.Y. (2025). Reactive oxygen species-responsive nanocomposite hydrogels for accurate drug delivery and localized PDT/PTT/chemo synergistic cancer therapy. Eur. Polym. J..

[32] Hu P., Wang W., Sha J., Xing Y., Wang Y., Wu C., Li J., Gao K., Dong H., Zheng S. (2023). Tumor microenvironment responsive-multifunctional nanocomposites knotted injectable hydrogels for enhanced synergistic chemodynamic and chemo-photothermal therapies. Mater. Des..

[33] Huang Z., Xiao H., Lu X., Yan W., Ji Z. (2018). Enhanced photo/chemo combination efficiency against bladder tumor by encapsulation of DOX and ZnPC into in situ-formed thermosensitive polymer hydrogel. Int. J. Nanomed..

[34] Rothe R., Xu Y., Wodtke J., Brandt F., Meister S., Laube M., Lollini P.-L., Zhang Y., Pietzsch J., Hauser S. (2024). Programmable Release of Chemotherapeutics from Ferrocene-Based Injectable Hydrogels Slows Melanoma Growth. Adv. Healthc. Mater..

[35] Zeng Q., Gong Y., Jiao W., Xu J., Chen X., Xu R., Liu Y., Liang X., Li G., Liu J. (2025). Smart ultra-long-lasting sequentially triggerable and artfully implantable nozzle system for on-demand drug delivery for chronotherapy. Sci. Adv..

[36] Bhat M.A., Rather R.A., Yaseen Z., Shalla A.H. (2022). Viscoelastic and smart swelling disposition of Carboxymethylcellulose based hydrogels substantiated by Gemini surfactant and in-vitro encapsulation and controlled release of Quercetin. Int. J. Biol. Macromol..

[37] Narayan R., Nayak U.Y., Raichur A.M., Garg S. (2018). Mesoporous Silica Nanoparticles: A Comprehensive Review on Synthesis and Recent Advances. Pharmaceutics.

[38] Liu F., Wang Z., Guo H., Li H., Chen Y., Guan S. (2023). A Double-Layer Hydrogel Dressing with High Mechanical Strength and Water Resistance Used for Drug Delivery. Molecules.

[39] Meena P., Singh P., Warkar S.G. (2023). Development and assessment of carboxymethyl tamarind kernel gum-based pH-responsive hydrogel for release of diclofenac sodium. Eur. Polym. J..

[40] Siddiqua A., Ranjha N.M., Rehman S., Shoukat H., Ramzan N., Sultana H. (2022). Preparation and characterization of methylene bisacrylamide crosslinked pectin/acrylamide hydrogels. Polym. Bull..

[41] Zhao J., Li S., Zhao Y., Peng Z. (2020). Effects of cellulose nanocrystal polymorphs and initial state of hydrogels on swelling and drug release behavior of alginate-based hydrogels. Polym. Bull..

[42] Gupta P., Vermani K., Garg S. (2002). Hydrogels: From controlled release to pH-responsive drug delivery. Drug Discov. Today.

[43] Sui B., Cheng C., Wang M., Hopkins E., Xu P. (2019). Heterotargeted Nanococktail with Traceless Linkers for Eradicating Cancer. Adv. Funct. Mater..

[44] Fang T., Cao X., Ibnat M., Chen G. (2022). Stimuli-responsive nanoformulations for CRISPR-Cas9 genome editing. J. Nanobiotechnol..

[45] Jiang Z., Zheng Z., Yu S., Gao Y., Ma J., Huang L., Yang L. (2023). Nanofiber Scaffolds as Drug Delivery Systems Promoting Wound Healing. Pharmaceutics.

[46] Gu W., Fan R., Quan J., Cheng Y., Wang S., Zhang H., Zheng A., Song S. (2022). Intracranial In Situ Thermosensitive Hydrogel Delivery of Temozolomide Accomplished by PLGA-PEG-PLGA Triblock Copolymer Blending for GBM Treatment. Polymers.

[47] Vieira I.R.S., Tessaro L., Lima A.K.O., Velloso I.P.S., Conte-Junior C.A. (2023). Recent Progress in Nanotechnology Improving the Therapeutic Potential of Polyphenols for Cancer. Nutrients.

[48] Zheng P., Wei Y., Cao K., Xu C., Yu S., Liu Y., Li M., Zhang C., Wang T. (2025). Sustained—Release microspheric gel of meloxicam: Preparation, evaluation in vitro and in vivo. Biomed. Microdevices.

[49] Alshehri A.M., Wilson O.C. (2024). Biomimetic Hydrogel Strategies for Cancer Therapy. Gels.

[50] Li L., Scheiger J.M., Levkin P.A. (2019). Design and Applications of Photoresponsive Hydrogels. Adv. Mater..

[51] Boedtkjer E., Pedersen S.F. (2020). The Acidic Tumor Microenvironment as a Driver of Cancer. Annu. Rev. Physiol..

[52] Ding H.M., Ma Y.Q. (2013). Controlling cellular uptake of nanoparticles with pH-sensitive polymers. Sci. Rep..

[53] Ye C., Li W.J., Yu H.H., Feng Q.P., Wu X., Zhu Y.T., Hu M.Y., Xiang S.Y., Yu S.Q. (2020). Binary blended co-delivery nanoparticles with the characteristics of precise pH-responsive acting on tumor microenvironment. Mater. Sci. Eng. C Mater. Biol. Appl..

[54] Andrade F., Roca-Melendres M.M., Durán-Lara E.F., Rafael D., Schwartz S. (2021). Stimuli-Responsive Hydrogels for Cancer Treatment: The Role of pH, Light, Ionic Strength and Magnetic Field. Cancers.

[55] Fu Z., Wang S., Zhou X., Ouyang L., Chen Z., Deng G. (2025). Harnessing the Power of Traditional Chinese Medicine in Cancer Treatment: The Role of Nanocarriers. Int. J. Nanomed..

[56] Ding H., Tan P., Fu S., Tian X., Zhang H., Ma X., Gu Z., Luo K. (2022). Preparation and application of pH-responsive drug delivery systems. J. Control. Release.

[57] Hughes K.A., Misra B., Maghareh M., Samart P., Nguyen E., Hussain S., Geldenhuys W.J., Bobbala S. (2024). Flash nanoprecipitation allows easy fabrication of pH-responsive acetalated dextran nanoparticles for intracellular release of payloads. Discov. Nano.

[58] Malek S., Mahmoudi A., Hashemi H., Tayebi R., Jaafari M.R., Mohammadi M., Malaekeh-Nikouei B. (2025). Innovative in-situ forming composite hydrogel: pH-responsive nanogel and combretastatin A4 liposome for breast cancer therapy. Carbohydr. Polym. Technol. Appl..

[59] Li J., Wang Z., Han H., Xu Z., Li S., Zhu Y., Chen Y., Ge L., Zhang Y. (2022). Short and simple peptide-based pH-sensitive hydrogel for antitumor drug delivery. Chin. Chem. Lett..

[60] Yang Y., Xie H., Zhou B., Liu Y., Hao J. (2026). pH-responsive dynamic organic nanocomposite hydrogels for enhanced local tumor combination therapy. J. Biomater. Appl..

[61] Qian X., Guan L., Shen L., Zhai C., Cheng Y., Pan G., Jiang Z. (2025). Recent advances in hydrogel-assisted treatment of malignant bone tumors. Mater. Today Bio.

[62] Kang J.H., Turabee M.H., Lee D.S., Kwon Y.J., Ko Y.T. (2021). Temperature and pH-responsive in situ hydrogels of gelatin derivatives to prevent the reoccurrence of brain tumor. Biomed. Pharmacother..

[63] Zhou J., Wang H., Chen H., Ling Y., Xi Z., Lv M., Chen J. (2023). pH-responsive nanocomposite hydrogel for simultaneous prevention of postoperative adhesion and tumor recurrence. Acta Biomater..

[64] Jiang P., Cheng Y., Yu S., Lu J., Wang H. (2018). Study on the Effect of 1-Butanol Soluble Lignin on Temperature-Sensitive Gel. Polymers.

[65] Yang J., Yao M.H., Jin R.M., Zhao D.H., Zhao Y.D., Liu B. (2017). Polypeptide-Engineered Hydrogel Coated Gold Nanorods for Targeted Drug Delivery and Chemo-photothermal Therapy. ACS Biomater. Sci. Eng..

[66] Zhang Y., Lu Y., Li S., Zheng F., Dong Y., Tang H., Wang X., Wang J. (2025). Precision theranostics in cervical Cancer: Harnessing stimuli-responsive hydrogels for tumor microenvironment-targeted therapy and diagnosis. Mater. Today Bio.

[67] Zhou W., Duan Z., Zhao J., Fu R., Zhu C., Fan D. (2022). Glucose and MMP-9 dual-responsive hydrogel with temperature sensitive self-adaptive shape and controlled drug release accelerates diabetic wound healing. Bioact. Mater..

[68] Wei W., Li H., Yin C., Tang F. (2020). Research progress in the application of in situ hydrogel system in tumor treatment. Drug Deliv..

[69] Tan W., Chen S., Xu Y., Chen M., Liao H., Niu C. (2023). Temperature-Sensitive Nanocarbon Hydrogel for Photothermal Therapy of Tumors. Int. J. Nanomed..

[70] Huo Y., Wang Q., Liu Y., Wang J., Li Q., Li Z., Dong Y., Huang Y., Wang L. (2019). A temperature-sensitive phase-change hydrogel of topotecan achieves a long-term sustained antitumor effect on retinoblastoma cells. Onco Targets Ther..

[71] Meng D., Lei H., Zheng X., Han Y., Sun R., Zhao D., Liu R. (2019). A temperature-sensitive phase-change hydrogel of tamoxifen achieves the long-acting antitumor activation on breast cancer cells. Onco Targets Ther..

[72] Li B., Criado-Gonzalez M., Adam A., Bizeau J., Mélart C., Carvalho A., Bégin S., Bégin D., Jierry L., Mertz D. (2022). Peptide Hydrogels Assembled from Enzyme-Adsorbed Mesoporous Silica Nanostructures for Thermoresponsive Doxorubicin Release. ACS Appl. Nano Mater..

[73] Li X.Y., Moesta A.K., Xiao C., Nakamura K., Casey M., Zhang H., Madore J., Lepletier A., Aguilera A.R., Sundarrajan A. (2019). Targeting CD39 in Cancer Reveals an Extracellular ATP- and Inflammasome-Driven Tumor Immunity. Cancer Discov..

[74] Xu X., Jiang Y., Lu C. (2022). Self-Assembled ATP-Responsive DNA Nanohydrogel for Specifically Activated Fluorescence Imaging and Chemotherapy in Cancer Cells. Anal. Chem..

[75] Wang D., Liu J., Duan J., Yi H., Liu J., Song H., Zhang Z., Shi J., Zhang K. (2023). Enrichment and sensing tumor cells by embedded immunomodulatory DNA hydrogel to inhibit postoperative tumor recurrence. Nat. Commun..

[76] Chen W.-H., Liao W.-C., Sohn Y.S., Fadeev M., Cecconello A., Nechushtai R., Willner I. (2018). Stimuli-Responsive Nucleic Acid-Based Polyacrylamide Hydrogel-Coated Metal–Organic Framework Nanoparticles for Controlled Drug Release. Adv. Funct. Mater..

[77] Sun L., Shen F., Tian L., Tao H., Xiong Z., Xu J., Liu Z. (2021). ATP-Responsive Smart Hydrogel Releasing Immune Adjuvant Synchronized with Repeated Chemotherapy or Radiotherapy to Boost Antitumor Immunity. Adv. Mater..

[78] Xiao L., Li Q., Huang Y., Fan Z., Ma L., Liu B., Yuan X. (2022). Construction of a Redox-Related Prognostic Model with Predictive Value in Survival and Therapeutic Response for Patients with Lung Adenocarcinoma. J. Healthc. Eng..

[79] Su X., Cao Y., Liu Y., Ouyang B., Ning B., Wang Y., Guo H., Pang Z., Shen S. (2021). Localized disruption of redox homeostasis boosting ferroptosis of tumor by hydrogel delivery system. Mater. Today Bio.

[80] Ballatori N., Krance S.M., Notenboom S., Shi S., Tieu K., Hammond C.L. (2009). Glutathione dysregulation and the etiology and progression of human diseases. Biol. Chem..

[81] Li L., Lei D., Zhang J., Xu L., Li J., Jin L., Pan L. (2022). Dual-Responsive Alginate Hydrogel Constructed by Sulfhdryl Dendrimer as an Intelligent System for Drug Delivery. Molecules.

[82] Zhang X., Shi K., Mao J., Mao K., Jia Y., Zhang J., Wang Q., Bai R., Gao F., Liu S. (2024). Ultra-strong penetrating and GSH-responsive oral drug delivery system improved therapeutic effect of gemcitabine for pancreatic tumors. Nano Today.

[83] Michalicha A., Belcarz A., Giannakoudakis D.A., Staniszewska M., Barczak M. (2024). Designing Composite Stimuli-Responsive Hydrogels for Wound Healing Applications: The State-of-the-Art and Recent Discoveries. Materials.

[84] El-Husseiny H.M., Mady E.A., Hamabe L., Abugomaa A., Shimada K., Yoshida T., Tanaka T., Yokoi A., Elbadawy M., Tanaka R. (2022). Smart/stimuli-responsive hydrogels: Cutting-edge platforms for tissue engineering and other biomedical applications. Mater. Today Bio.

[85] Conte R., Valentino A., Romano S., Margarucci S., Petillo O., Calarco A. (2024). Stimuli-Responsive Nanocomposite Hydrogels for Oral Diseases. Gels.

[86] Lin Z., Zhao Z., Lin X., Yang Z., Wang L., Xi R., Long D. (2025). Advances in oral treatment of inflammatory bowel disease using protein-based nanoparticle drug delivery systems. Drug Deliv..

[87] Su D., Zhang D. (2021). Linker Design Impacts Antibody-Drug Conjugate Pharmacokinetics and Efficacy via Modulating the Stability and Payload Release Efficiency. Front. Pharmacol..

[88] Mei L., Mei Q., Dong W., Wu S. (2024). Redox-responsive self-assembled peptide hydrogel for mitochondrial-targeted anticancer drug delivery. Appl. Mater. Today.

[89] Chu C.W., Cheng W.J., Wen B.Y., Liang Y.K., Sheu M.T., Chen L.C., Lin H.L. (2024). Preparation and Rheological Evaluation of Thiol-Maleimide/Thiol-Thiol Double Self-Crosslinking Hyaluronic Acid-Based Hydrogels as Dermal Fillers for Aesthetic Medicine. Gels.

[90] Zhu Y., Wang L., Li Y., Huang Z., Luo S., He Y., Han H., Raza F., Wu J., Ge L. (2020). Injectable pH and redox dual responsive hydrogels based on self-assembled peptides for anti-tumor drug delivery. Biomater. Sci..

[91] Kasiński A., Zielińska-Pisklak M., Oledzka E., Sobczak M. (2020). Smart Hydrogels—Synthetic Stimuli-Responsive Antitumor Drug Release Systems. Int. J. Nanomed..

[92] Zhang Y., Xu Y., Wei C., Zhang Y., Yang L., Song Z., Lang M. (2017). Diselenide-containing poly(ε-caprolactone)-based thermo-responsive hydrogels with oxidation and reduction-triggered degradation. Mater. Today Chem..

[93] Li K., Huang Y., Xiu C. (2025). Tumor microenvironment-activated ROS enhancers for effective inhibition of osteosarcoma. BMC Biotechnol..

[94] Zhang L., Fan Y., Yang Z., Yang M., Wong C.Y. (2021). NIR-II-driven and glutathione depletion-enhanced hypoxia-irrelevant free radical nanogenerator for combined cancer therapy. J. Nanobiotechnol..

[95] Wang H.Q., Man Q.W., Huo F.Y., Gao X., Lin H., Li S.R., Wang J., Su F.C., Cai L., Shi Y. (2022). STAT3 pathway in cancers: Past, present, and future. MedComm.

[96] Wang X., Zhang X., Xu J., Xu S., Huang K., Ni Q., Shen X., Zhang W., Liu T., Dong T. (2025). High-concentration L-methionine as a potent antioxidant for oxidation resistance and stability enhancement in high-concentration antibody therapeutics. Int. J. Pharm. X.

[97] Li F., Ding J., Li Z., Rong Y., He C., Chen X. (2024). ROS-responsive thermosensitive polypeptide hydrogels for localized drug delivery and improved tumor chemoimmunotherapy. Biomater. Sci..

[98] Zou L., Hou Y., Nie X., Wang S., Tian S., Sun Z., Sun Z., Xu X., Li G., Ma G. (2025). All-Small-Molecule Supramolecular Hydrogel Combining Self-Delivery and ROS-Responsive Release for Inhibiting Tumor Growth and Postoperative Recurrence. ACS Appl. Mater. Interfaces.

[99] Li Z., Xu W., Yang J., Wang J., Wang J., Zhu G., Li D., Ding J., Sun T. (2022). A Tumor Microenvironments-Adapted Polypeptide Hydrogel/Nanogel Composite Boosts Antitumor Molecularly Targeted Inhibition and Immunoactivation. Adv. Mater..

[100] Hu K., Liang L., Song J. (2025). Development of a ROS-responsive, glutathione-functionalized injectable hydrogel system for controlled drug release. J. Biomater. Appl..

[101] Saadat M., Taherian A.A., Aldaghi M.R., Raise-Abdullahi P., Sameni H.R., Vafaei A.A. (2023). *Prangos ferulacea* (L.) ameliorates behavioral alterations, hippocampal oxidative stress markers, and apoptotic deficits in a rat model of autism induced by valproic acid. Brain Behav..

[102] Van Gheluwe L., Chourpa I., Gaigne C., Munnier E. (2021). Polymer-Based Smart Drug Delivery Systems for Skin Application and Demonstration of Stimuli-Responsiveness. Polymers.

[103] Ruan J., Liu H., Chen B., Wang F., Wang W., Zha Z., Qian H., Miao Z., Sun J., Tian T. (2021). Interfacially Engineered Zn*_x_*Mn_1−*x*_S@Polydopamine Hollow Nanospheres for Glutathione Depleting Photothermally Enhanced Chemodynamic Therapy. ACS Nano.

[104] Ma W., Wang X., Zhang D., Mu X. (2024). Research Progress of Disulfide Bond Based Tumor Microenvironment Targeted Drug Delivery System. Int. J. Nanomed..

[105] Zha J., Mao X., Hu S., Shang K., Yin J. (2021). Acid- and Thiol-Cleavable Multifunctional Codelivery Hydrogel: Fabrication and Investigation of Antimicrobial and Anticancer Properties. ACS Appl. Bio Mater..

[106] Liu H., Deng Z., Li T., Bu J., Wang D., Wang J., Liu M., Li J., Yang Y., Zhong S. (2022). Fabrication, GSH-responsive drug release, and anticancer properties of thioctic acid-based intelligent hydrogels. Colloids Surf. B Biointerfaces.

[107] Li X., Shi Y., Xu S. (2022). Local delivery of tumor-targeting nano-micelles harboring GSH-responsive drug release to improve antitumor efficiency. Polym. Adv. Technol..

[108] Zhang X., Li Y., Zhang Y., Wang S., Zhao J., Wang T. (2025). Glutathione depletion-based pH-responsive injectable hydrogels for synergistic treatment of colon tumor. Int. J. Biol. Macromol..

[109] Ning S., Mo J., Huang R., Liu B., Fu B., Ding S., Yang H., Cui Y., Yao L. (2023). Injectable thermo-sensitive hydrogel loaded hollow copper sulfide nanoparticles for ROS burst in TME and effective tumor treatment. Front. Bioeng. Biotechnol..

[110] Lv X., Liu Z., Qi P., Chen K. (2025). Thermosensitive hydrogel loaded with nanozyme and BPTES for enhanced tumor catalytic therapy. Colloids Surf. B Biointerfaces.

[111] Noddeland H.K., Lind M., Jensen L.B., Petersson K., Skak-Nielsen T., Larsen F.H., Malmsten M., Heinz A. (2023). Design and characterization of matrix metalloproteinase-responsive hydrogels for the treatment of inflammatory skin diseases. Acta Biomater..

[112] Sobczak M. (2022). Enzyme-Responsive Hydrogels as Potential Drug Delivery Systems-State of Knowledge and Future Prospects. Int. J. Mol. Sci..

[113] Chandrawati R. (2016). Enzyme-responsive polymer hydrogels for therapeutic delivery. Exp. Biol. Med..

[114] Wang W., Wu X., Yuan D., Shi J. (2025). Enzyme-Responsive Metallopeptide Hydrogel Enables Cancer Cell-Selective Prodrug Activation via Bioorthogonal Catalysis. Small.

[115] Chen X., Liu Z., Parker S.G., Zhang X., Gooding J.J., Ru Y., Liu Y., Zhou Y. (2016). Light-Induced Hydrogel Based on Tumor-Targeting Mesoporous Silica Nanoparticles as a Theranostic Platform for Sustained Cancer Treatment. ACS Appl. Mater. Interfaces.

[116] Barbugian F., Salerno D., Ballarini E., Crippa L., Francesconi O., Mantegazza F., Cavaletti G., Roelens S., Leone G., Pepi S. (2025). Bioresponsive Hyaluronic Acid-Based Hydrogel Inhibits Matrix Metalloproteinase-2 in Glioblastoma Microenvironment. ChemMedChem.

[117] Liu J., Xiao S., Huang C., Zhang X., Cai R., Qu S., Peng Y., Xie L. (2025). Emodin nanoparticles-loaded injectable hydrogel with deep tumor penetration for triple-negative breast cancer therapy. Biomater. Adv..

[118] Liu T., Yao Y., Zhang S., Liang Y., Zhou Y., Gai Y., Cai Y., Zhou C., Zhang B., Wang Y. (2026). A hyaluronidase detection platform combined with enzyme-responsive hydrogel and periodic magnetic-modulated electrochemical analysis system. Sens. Actuators B Chem..

[119] Yoshisaki R., Kimura S., Yokoya M., Yamanaka M. (2021). Enzymatic Hydrolysis-Responsive Supramolecular Hydrogels Composed of Maltose-Coupled Amphiphilic Ureas. Chem. Asian J..

[120] Li Y., Lin D., Chen J., Zhang W., Jin H., Feng S., Mao H., Hou J., Li X., Wang Y. (2025). Alkaline phosphatase-responsive hydrogel for efficient management of autoimmune intraocular inflammation. J. Control. Release.

[121] Jia X., Dong Y., Lu J., Yang Z., Xu R., Zhang X., Jiao J., Zhang Z., Lin Y., Chu F. (2025). A self-assembly enzyme-like hydrogel with ROS scavenging and immunomodulatory capability for microenvironment-responsive wound healing acceleration. Int. J. Pharm..

[122] Agarwal V., Varshney N., Singh S., Kumar N., Chakraborty A., Sharma B., Jha H.C., Sarma T.K. (2023). Cobalt-Adenosine Monophosphate Supramolecular Hydrogel with pH-Responsive Multi-Nanozymatic Activity. ACS Appl. Bio Mater..

[123] Gao Y., Luo Q., Qiao S., Wang L., Dong Z., Xu J., Liu J. (2014). Enzymetically regulating the self-healing of protein hydrogels with high healing efficiency. Angew. Chem. Int. Ed. Engl..

[124] Jafari H., Alimoradi H., Delporte C., Bernaerts K.V., Heidari R., Podstawczyk D., Niknezhad S.V., Shavandi A. (2022). An injectable, self-healing, 3D printable, double network co-enzymatically crosslinked hydrogel using marine poly- and oligo-saccharides for wound healing application. Appl. Mater. Today.

[125] Rapp T.L., DeForest C.A. (2023). Tricolor visible wavelength-selective photodegradable hydrogel biomaterials. Nat. Commun..

[126] Jervis P.J., Hilliou L., Pereira R.B., Pereira D.M., Martins J.A., Ferreira P.M.T. (2021). Evaluation of a Model Photo-Caged Dehydropeptide as a Stimuli-Responsive Supramolecular Hydrogel. Nanomaterials.

[127] Xing Y., Zeng B., Yang W. (2022). Light responsive hydrogels for controlled drug delivery. Front. Bioeng. Biotechnol..

[128] Kopyeva I., Brady R.P., DeForest C.A. (2025). Light-based fabrication and 4D customization of hydrogel biomaterials. Nat. Rev. Bioeng..

[129] Huang F., Liu B., Guo Y., Yang Z., Li S., Chen Z., Qu S. (2026). Photodegradable hydrogels: Connecting network evolution and material properties by a photo-chemo-mechanical coupling model. Mater. Sci. Eng. R Rep..

[130] Zhao D., Tang Q., Zhou Q., Peng K., Yang H., Zhang X. (2018). A photo-degradable injectable self-healing hydrogel based on star poly(ethylene glycol)-b-polypeptide as a potential pharmaceuticals delivery carrier. Soft Matter.

[131] Abbas M., Xing R., Zhang N., Zou Q., Yan X. (2018). Antitumor Photodynamic Therapy Based on Dipeptide Fibrous Hydrogels with Incorporation of Photosensitive Drugs. ACS Biomater. Sci. Eng..

[132] Zheng Y., Wu Q., Zhu Q., Ren A., Guo L., Huang S., Huang Z.-S., Li Q., Li Y., Chen C. (2025). Thermosensitive hydrogel as an injectable aggregation-induced emission photosensitizer delivery scaffold for lung cancer therapeutics with long-acting photodynamic therapy. Dye. Pigment..

[133] Shu G., Zhu W., Jiang Y., Li X., Pan J., Zhang X., Zhang X., Sun S.-K. (2021). Persistent Luminescence Immune Hydrogel for Photodynamic-Immunotherapy of Tumors In Vivo. Adv. Funct. Mater..

[134] Tang L., Zhang A., Zhang Z., Zhao Q., Li J., Mei Y., Yin Y., Wang W. (2022). Multifunctional inorganic nanomaterials for cancer photoimmunotherapy. Cancer Commun..

[135] Chen G., Ullah A., Xu G., Xu Z., Wang F., Liu T., Su Y., Zhang T., Wang K. (2021). Topically applied liposome-in-hydrogels for systematically targeted tumor photothermal therapy. Drug Deliv..

[136] Higashi B., Mariano T.B., de Abreu Filho B.A., Gonçalves R.A.C., de Oliveira A.J.B. (2020). Effects of fructans and probiotics on the inhibition of Klebsiella oxytoca and the production of short-chain fatty acids assessed by NMR spectroscopy. Carbohydr. Polym..

[137] Zhao Y., Wang W., Liu M., Cai Y., Wang Y., Dong Y., Bai Y.K., Zhu J., Tay F.R., Niu L. (2026). Mn_3_O_4_-potentiated bifunctional hydrogel for mild temperature-controlled tumor ablation and osteogenesis. Bioact. Mater..

[138] Sun J., Guo Y., Xing R., Jiao T., Zou Q., Yan X. (2017). Synergistic in vivo photodynamic and photothermal antitumor therapy based on collagen-gold hybrid hydrogels with inclusion of photosensitive drugs. Colloids Surf. A Physicochem. Eng. Asp..

[139] Zhang Y., Zhu C., Zhang Z., Zhao J., Yuan Y., Wang S. (2021). Oxidation triggered formation of polydopamine-modified carboxymethyl cellulose hydrogel for anti-recurrence of tumor. Colloids Surf. B Biointerfaces.

[140] Zhang L., Li Q., Liu J., Deng Z., Zhang X., Alifu N., Zhang X., Yu Z., Liu Y., Lan Z. (2024). Recent advances in functionalized ferrite nanoparticles: From fundamentals to magnetic hyperthermia cancer therapy. Colloids Surf. B Biointerfaces.

[141] Chen X., Wang H., Shi J., Chen Z., Wang Y., Gu S., Fu Y., Huang J., Ding J., Yu L. (2023). An injectable and active hydrogel induces mutually enhanced mild magnetic hyperthermia and ferroptosis. Biomaterials.

[142] Chao Y., Chen G., Liang C., Xu J., Dong Z., Han X., Wang C., Liu Z. (2019). Iron Nanoparticles for Low-Power Local Magnetic Hyperthermia in Combination with Immune Checkpoint Blockade for Systemic Antitumor Therapy. Nano Lett..

[143] Qian K.Y., Song Y., Yan X., Dong L., Xue J., Xu Y., Wang B., Cao B., Hou Q., Peng W. (2020). Injectable ferrimagnetic silk fibroin hydrogel for magnetic hyperthermia ablation of deep tumor. Biomaterials.

[144] Xie G., Wang L., Li B., Zhang C., Zhang X. (2023). Transform commercial magnetic materials into injectable gel for magnetic hyperthermia therapy in vivo. Colloids Surf. B Biointerfaces.

[145] Zuo X., Tang H., Zhu X., Zhang D., Gao W. (2021). Injectable magnetic hydrogels for self-regulating magnetic hyperthermia and drug release. Mod. Phys. Lett. B.

[146] Yin P., Brozovic A., Zhang W., Wu C. (2026). Core-shell hydrogel microspheres with sequential drug release and magnetothermal synergy for drug-resistant ovarian cancer. Biomater. Sci..

[147] Yang H., Li X., Yu Y., Li Q., Zheng Y., Xia D. (2025). Ultrasound-responsive hydrogels for bone and cartilage tissue engineering. Mater. Today Bio.

[148] Zhang F., Lv M., Wang S., Li M., Wang Y., Hu C., Hu W., Wang X., Wang X., Liu Z. (2024). Ultrasound-triggered biomimetic ultrashort peptide nanofiber hydrogels promote bone regeneration by modulating macrophage and the osteogenic immune microenvironment. Bioact. Mater..

[149] Sun Y., Chen L.G., Fan X.M., Pang J.L. (2022). Ultrasound Responsive Smart Implantable Hydrogels for Targeted Delivery of Drugs: Reviewing Current Practices. Int. J. Nanomed..

[150] Seynhaeve A.L.B., Amin M., Haemmerich D., van Rhoon G.C., Ten Hagen T.L.M. (2020). Hyperthermia and smart drug delivery systems for solid tumor therapy. Adv. Drug Deliv. Rev..

[151] Meng Z., Zhang Y., She J., Zhou X., Xu J., Han X., Wang C., Zhu M., Liu Z. (2021). Ultrasound-Mediated Remotely Controlled Nanovaccine Delivery for Tumor Vaccination and Individualized Cancer Immunotherapy. Nano Lett..

[152] Wu C.H., Sun M.K., Shieh J., Chen C.S., Huang C.W., Dai C.A., Chang S.W., Chen W.S., Young T.H. (2018). Ultrasound-responsive NIPAM-based hydrogels with tunable profile of controlled release of large molecules. Ultrasonics.

[153] Liu S., Sun M., Fan Z. (2025). Ultrasonic-Responsive Pluronic P105/F127 Nanogels for Overcoming Multidrug Resistance in Cancer. Gels.

[154] Nowak K.M., Schwartz M.R., Breza V.R., Price R.J. (2022). Sonodynamic therapy: Rapid progress and new opportunities for non-invasive tumor cell killing with sound. Cancer Lett..

[155] Wang P., Chen J., Zhong R., Xia Y., Wu Z., Zhang C., Yao H. (2024). Recent advances of ultrasound-responsive nanosystems in tumor immunotherapy. Eur. J. Pharm. Biopharm..

[156] Ma X., Sekhar K.P.C., Zhang P., Cui J. (2024). Advances in stimuli-responsive injectable hydrogels for biomedical applications. Biomater. Sci..

[157] Yi Q., Ma J., Kang K., Gu Z. (2016). Dual cellular stimuli-responsive hydrogel nanocapsules for delivery of anticancer drugs. J. Mater. Chem. B.

[158] Massoumi B., Mossavi R., Motamedi S., Derakhshankhah H., Vandghanooni S., Jaymand M. (2021). Fabrication of a dual stimuli-responsive magnetic nanohydrogel for delivery of anticancer drugs. Drug Dev. Ind. Pharm..

[159] Kim H.U., Choi D.G., Lee H., Shim M.S., Bong K.W. (2018). Fabrication of dual stimuli-responsive multicompartmental drug carriers for tumor-selective drug release. Lab Chip.

[160] Zeng Y., Zhang C., Du D., Li Y., Sun L., Han Y., He X., Dai J., Shi L. (2022). Metal-organic framework-based hydrogel with structurally dynamic properties as a stimuli-responsive localized drug delivery system for cancer therapy. Acta Biomater..

[161] Kuddushi M., Ray D., Aswal V., Hoskins C., Malek N. (2020). Poly(vinyl alcohol) and Functionalized Ionic Liquid-Based Smart Hydrogels for Doxorubicin Release. ACS Appl. Bio Mater..

[162] Gao J., Li M., Chen H., Xu Z., Li J., Kong Y., Zuo X. (2025). Synthesis of stimuli-responsive copolymeric hydrogels for temperature, reduction and pH-controlled drug delivery. J. Ind. Eng. Chem..

[163] Jahanban-Esfahlan R., Soleimani K., Derakhshankhah H., Haghshenas B., Rezaei A., Massoumi B., Farnudiyan-Habibi A., Samadian H., Jaymand M. (2021). Multi-stimuli-responsive magnetic hydrogel based on Tragacanth gum as a de novo nanosystem for targeted chemo/hyperthermia treatment of cancer. J. Mater. Res..

[164] Dai Z., Zhang Q., Li X., Chen Q., Chen J., Wang M., Chen H. (2023). In situ forming pH/ROS-responsive niche-like hydrogel for ultrasound-mediated multiple therapy in synergy with potentiating anti-tumor immunity. Mater. Today.

[165] Rasib S.Z.M., Ahmad Z., Khan A., Akil H.M., Othman M.B.H., Hamid Z.A.A., Ullah F. (2018). Synthesis and evaluation on pH- and temperature-responsive chitosan-p(MAA-co-NIPAM) hydrogels. Int. J. Biol. Macromol..

[166] Liao W.-C., Lilienthal S., Kahn J.S., Riutin M., Sohn Y.S., Nechushtai R., Willner I. (2017). pH- and ligand-induced release of loads from DNA–acrylamide hydrogel microcapsules. Chem. Sci..

[167] Solanki R., Bhatia D. (2024). Stimulus-Responsive Hydrogels for Targeted Cancer Therapy. Gels.

[168] Hu X., Wang Y., Zhang L., Xu M., Dong W., Zhang J. (2017). Redox/pH dual stimuli-responsive degradable Salecan-g-SS-poly(IA-co-HEMA) hydrogel for release of doxorubicin. Carbohydr. Polym..

[169] Yang W.J., Zhou P., Liang L., Cao Y., Qiao J., Li X., Teng Z., Wang L. (2018). Nanogel-Incorporated Injectable Hydrogel for Synergistic Therapy Based on Sequential Local Delivery of Combretastatin-A4 Phosphate (CA4P) and Doxorubicin (DOX). ACS Appl. Mater. Interfaces.

[170] Guan Q., Hu T., Zhang L., Yu M., Niu J., Ding Z., Yu P., Yuan G., An Z., Pei J. (2024). Concerting magnesium implant degradation facilitates local chemotherapy in tumor-associated bone defect. Bioact. Mater..

[171] Yang B., Wang K., Zhang D., Ji B., Zhao D., Wang X., Zhang H., Kan Q., He Z., Sun J. (2019). Polydopamine-modified ROS-responsive prodrug nanoplatform with enhanced stability for precise treatment of breast cancer. RSC Adv..

[172] Li Z., He P., Long Y., Yuan G., Shen W., Chen Z., Zhang B., Wang Y., Yue D., Seidl C. (2021). Drug Repurposing of Pantoprazole and Vitamin C Targeting Tumor Microenvironment Conditions Improves Anticancer Effect in Metastatic Castration-Resistant Prostate Cancer. Front. Oncol..

[173] Wang P., Gong Q., Hu J., Li X., Zhang X. (2021). Reactive Oxygen Species (ROS)-Responsive Prodrugs, Probes, and Theranostic Prodrugs: Applications in the ROS-Related Diseases. J. Med. Chem..

[174] Lee Y.M., Lu Z.W., Wu Y.C., Liao Y.J., Kuo C.Y. (2024). An injectable, chitosan-based hydrogel prepared by Schiff base reaction for anti-bacterial and sustained release applications. Int. J. Biol. Macromol..

[175] Yi D., Wang T., Xu S., Liu Y., Wang Z., Li N., Wu Y., He Y. (2026). Injectable ROS/pH-Responsive Hydrogel for Synergistic Chemo-Photodynamic Tumor Therapy and Enhanced Wound Healing. Adv. Healthc. Mater..

[176] Cheng X., Jin Y., Sun T., Qi R., Li H., Fan W. (2016). An injectable, dual pH and oxidation-responsive supramolecular hydrogel for controlled dual drug delivery. Colloids Surf. B Biointerfaces.

[177] Dong A., Huang S., Qian Z., Xu S., Yuan W., Wang B. (2023). A pH-responsive supramolecular hydrogel encapsulating a CuMnS nanoenzyme catalyst for synergistic photothermal-photodynamic-chemodynamic therapy of tumours. J. Mater. Chem. B.

[178] Wang L., Yin Q., Liu C., Tang Y., Sun C., Zhuang J. (2021). Nanoformulations of Ursolic Acid: A Modern Natural Anticancer Molecule. Front. Pharmacol..

[179] Xue Y., Xia X., Yu B., Tao L., Wang Q., Huang S.W., Yu F. (2017). Selenylsulfide Bond-Launched Reduction-Responsive Superparamagnetic Nanogel Combined of Acid-Responsiveness for Achievement of Efficient Therapy with Low Side Effect. ACS Appl. Mater. Interfaces.

[180] Li W.Y., Wan J.J., Kan J.L., Wang B., Song T., Guan Q., Zhou L.L., Li Y.A., Dong Y.B. (2023). A biodegradable covalent organic framework for synergistic tumor therapy. Chem. Sci..

[181] Wang L., Zhou W., Wang Q., Xu C., Tang Q., Yang H. (2018). An Injectable, Dual Responsive, and Self-Healing Hydrogel Based on Oxidized Sodium Alginate and Hydrazide-Modified Poly(ethyleneglycol). Molecules.

[182] Chen Y., Zhang R., Zheng B., Cai C., Chen Z., Li H., Liu H. (2020). A Biocompatible, Stimuli-Responsive, and Injectable Hydrogel with Triple Dynamic Bonds. Molecules.

[183] Zhang M., Hu W., Cai C., Wu Y., Li J., Dong S. (2022). Advanced application of stimuli-responsive drug delivery system for inflammatory arthritis treatment. Mater. Today Bio.

[184] Hoang Thi T.T., Sinh L.H., Huynh D.P., Nguyen D.H., Huynh C. (2020). Self-Assemblable Polymer Smart-Blocks for Temperature-Induced Injectable Hydrogel in Biomedical Applications. Front. Chem..

[185] Li Y., Wang J., Liu S. (2025). Synthesis of Temperature/pH Dual-Responsive Double-Crosslinked Hydrogel on Medical Titanium Alloy Surface. Gels.

[186] Xie Y., Liu M., Cai C., Ye C., Guo T., Yang K., Xiao H., Tang X., Liu H. (2023). Recent progress of hydrogel-based local drug delivery systems for postoperative radiotherapy. Front. Oncol..

[187] Zhang M., Zhu C. (2024). Dynamic Hydrogels against Infections: From Design to Applications. Gels.

[188] Lin Z., Ding J., Chen X., He C. (2023). pH- and Temperature-responsive Hydrogels Based on Tertiary Amine-modified Polypeptides for Stimuli-responsive Drug Delivery. Chem. Asian J..

[189] Jommanee N., Chanthad C., Manokruang K. (2018). Preparation of injectable hydrogels from temperature and pH responsive grafted chitosan with tuned gelation temperature suitable for tumor acidic environment. Carbohydr. Polym..

[190] Tu L., Liao Z., Luo Z., Wu Y.L., Herrmann A., Huo S. (2021). Ultrasound-controlled drug release and drug activation for cancer therapy. Exploration.

[191] Guo Y., He X., Williams G.R., Zhou Y., Liao X., Xiao Z., Yu C., Liu Y. (2024). Tumor microenvironment-responsive hyperbranched polymers for controlled drug delivery. J. Pharm. Anal..

[192] Kumar N., Singh S., Sharma P., Kumar B., Kumar A. (2024). Single-, Dual-, and Multi-Stimuli-Responsive Nanogels for Biomedical Applications. Gels.

